# Enigmatic role of auxin response factors in plant growth and stress tolerance

**DOI:** 10.3389/fpls.2024.1398818

**Published:** 2024-06-10

**Authors:** Ling Liu, Baba Salifu Yahaya, Jing Li, Fengkai Wu

**Affiliations:** ^1^Faculty of Agriculture, Forestry and Food Engineering, Yibin University, Yibin, Sichuan, China; ^2^Maize Research Institute, Sichuan Agricultural University, Wenjiang, Sichuan, China; ^3^Key Laboratory of Biology and Genetic Improvement of Maize in Southwest Region, Ministry of Agriculture, Wenjiang, Sichuan, China

**Keywords:** ARF, Aux/IAA, growth and development, abiotic stresses, regulatory mechanisms

## Abstract

Abiotic and biotic stresses globally constrain plant growth and impede the optimization of crop productivity. The phytohormone auxin is involved in nearly every aspect of plant development. Auxin acts as a chemical messenger that influences gene expression through a short nuclear pathway, mediated by a family of specific DNA-binding transcription factors known as Auxin Response Factors (ARFs). ARFs thus act as effectors of auxin response and translate chemical signals into the regulation of auxin responsive genes. Since the initial discovery of the first ARF in Arabidopsis, advancements in genetics, biochemistry, genomics, and structural biology have facilitated the development of models elucidating ARF action and their contributions to generating specific auxin responses. Yet, significant gaps persist in our understanding of ARF transcription factors despite these endeavors. Unraveling the functional roles of ARFs in regulating stress response, alongside elucidating their genetic and molecular mechanisms, is still in its nascent phase. Here, we review recent research outcomes on ARFs, detailing their involvement in regulating leaf, flower, and root organogenesis and development, as well as stress responses and their corresponding regulatory mechanisms: including gene expression patterns, functional characterization, transcriptional, post-transcriptional and post- translational regulation across diverse stress conditions. Furthermore, we delineate unresolved questions and forthcoming challenges in ARF research.

## 1 Introduction

Plants face numerous abiotic and biotic stresses due to their sessile nature, including water and nutrient deficiencies, high salinity, extreme temperatures, radiation, heavy metal toxicity, and biotic infections. An estimated 90% of global arable lands are exposed to one or more of the above abiotic stresses (Dos Reis et al., 2012), projected to cause up to 70% yield loss in major crops (Mantri et al., 2012). The biotic stress caused by viral, fungal, and bacterial infections cause reduction in level of photosynthesis in all major crops and is the major cause of pre- and post-harvest losses. Biotic stresses are responsible for approximately, 28.2%, 37.4%, 31.2%, 40.3%, 26.3%, and 28.8% yield losses in wheat, rice, maize, potatoes, soybeans, and cotton, respectively (Wang et al., 2013). Adaptation to such stresses is crucial for optimizing performance of plants and stability of their successive generations. Developing stress-tolerant plants remains the ultimate goal of plant breeders due to their superior yields and stability (Kambona et al., 2023). Genetic manipulation of plants remains the most prominent approach to alleviating poverty, due to its potential to increasing crop yield and mitigating nutrient deficiencies, enabling the cultivation of salt affected lands, overcoming energy crisis and production of cost-efficient biopharmaceuticals using plants as cellular factories (Ahmad and Mukhtar, 2017). Additionally, genetic modification offers the possibility of identifying candidate genes, miRNAs and transcription factors (TFs) that participate in regulating specific plant processes to improve tolerance to abiotic stresses and enhance productivity. For example, overexpression of *McWRKY57* conferred tolerance to drought stress in *Arabidopsis* (Bai et al., 2023). miRNAq and nuclear factor YA8 enhanced salt tolerance by activating PEROSIDASE expression in response to reactive oxygen species (ROS) (Xing et al., 2021).

Plants have evolved intricate stress response mechanisms, including proper perception, signal transduction and respective physiological adjustments informed by the kind and duration of stress (Kranner et al., 2010). The perception of stress cues in plant is a complex network of input signals integrated in signal pathways that target regulators of plant growth and physiology (Scheres and van der Putten, 2017). Transcription regulation of stress-responsive genes is a pivotal biological process that confers stress tolerance in plants, and allows plants to strictly define and sustain their cellular identity and coordinates cellular activity during its life cycle (Casamassimi and Ciccodicola, 2019). Such regulations are mainly mediated by the temporal and spatial functioning of TFs that contain highly conserved DNA-binding domains (DBDs), with which they bind to specific DNA sequences in promoters of their target genes (Wang G. et al., 2015). On the other hand, TFs are either upregulated or downregulated by kinases or phosphatases and inturn binds to cis-regulatory elements in promoter of stress-inducible genes to enhance or suppress their transcription (Baillo et al., 2019). TFs also regulate stress induced responses in plants through mechanisms like posttranslational and epigenetic modifications such as variable nucleosome distribution, histone modification, DNA methylation, and synthesis of non-protein-coding RNAs (npcRNAs).

## 2 Molecular structure and classification of ARF proteins

Recent studies have traced the evolutionary origins of ARFs back to early charophyte algae, where a single proto-ARF gene existed (Mutte et al., 2018). Following an initial duplication event, proto-ARFs diversified into two classes (A/B and C) during the late-divergence charophytes. In the transition to land plants, a subsequent division of class A/B into distinct classes A and B established the three evolutionary classes recognized today: A, B, and C. Further duplications within these classes expanded and diversified the ARF family in higher land plants (Mutte et al., 2018). Since the identification of the first ARF (ARF1) in Arabidopsis, 22 more ARFs have been identified and characterized from the Arabidopsis genome (Moller et al., 2017). Homology cloning and genetic approaches have since been employed ino identifying numerous homologous ARF genes in various plant species after the release of genomic data and development of bioinformatics analyses. The 23 ARFs in Arabidopsis canbe divided into three subclasses: A, B, and C (Finet et al., 2013). Most ARFs possess similar topology, with three conserved protein domains, whose properties must be understood in details. Majority of ARFs generally contain a conserved N-terminus DNA-binding domain (DBD), a variable middle region (MR) that functions as either an activator or repressor domain and a conserved C-terminal dimerization domain (CTD), which is involved in protein-protein interactions (Dinesh et al., 2015). The functions and properties of each of these domains are enumerated below.

### 2.1 The DNA-binding domain of ARFs

Transcription factors are universal master regulators of gene expression that bind to unique DNA sequences in the promoter of their target genes to regulate their expression (Suter, 2020). A critical, yet unresolved in aspect of auxin biology is the mechanism by which the simple tryptophan-like indole-3-acetic acid triggers a wide range of cellular responses. During the last step of auxin signaling prior to gene regulation, the ARFs confer specificity to auxin response through selection of target genes. ARF TFs possess typical B3 DBD at their N-terminus, which allows them to bind to DNA motifs called Auxin Response Elements (AuxREs) (Boer et al., 2014; Weijers and Wagner, 2016). The first AuxRE was identified in pea (Ballas et al., 1993) and soybean (Ulmasov et al., 1995) in the promoters of auxin-responsive genes as TGTCTC (Liu et al., 1994). The identification of AuxRE is one of the most significant events that has enhanced the understanding of auxin-mediated regulation of gene expression and the creation of auxin-reporter systems (Hagen and Guilfoyle, 2002), and the identification of the first ARF protein (Ulmasov et al., 1997). The crystal structures of the DBD of ARF1 and ARF5/MONOPTEROS (MP) homodimers, as well as complex of ARF1 DBD with DNA has permitted visualization of protein-DNA interaction (Roosjen et al., 2018), and depicts how amino acids in the DBD interact with the DNA-binding motif TGTCTC (Freire-Rios et al., 2020). The higher affinity of ARFs to the TGTCGG element is because of deeper rotation of H136 into the major DNA groove, which forms additional hydrogen bonds with G5 and G6 in the TGTCGG structure (Boer et al., 2014; Freire-Rios et al., 2020). Mutations in these DNA-interacting amino acids interfered with the DNA binding properties of these ARFs and their biological functions. The TGTC serves as the invariable core element crucial for auxin response, while the final two nucleotides are variable (Boer et al., 2014). In recent years, adoption of advanced techniques has contributed to the identification of other AuxREs and the revelation that variation in the last two nucleotides of an AuxRE were permitted and could play a role in the affinity of ARFs for DNA binding. Although TGTCTC DNA-binding motif was the first to be identified, protein-binding microarrays (PBMs) has revealed that TGTCGG motif possesses relatively higher ARF binding affinity than the TGTCTC motif (Boer et al., 2014). It has been revealed through a ‘cistrome’ analysis that ARF2 and ARF5/MP have higher affinity for TGTCGG than the classical TGTCTC (O’Malley et al., 2016).

Through crystal structures, *in vitro*, and heterologous studies, a model in which ARF dimers bind with high affinity to distinct repeats of canonical AuxRE motifs has been unraveled. Like all TFs, ARFs bind to DNA as dimers and can homodimerize through their DBD by binding to tandem repeat motifs of TGTCNN elements. Configurations of the tandem repeat and the number of bases between the individual motifs determine their nomenclature: Inverted repeats (IR) where two AuxREs are oriented towards each other in different strands of DNA, direct repeat (DR) where two AuxREs follow each other in the same DNA strand and everted repeat (ER) where two AuxREs orient back to back in different strands of DNA (Freire-Rios et al., 2020). Yeast synthetic auxin signaling system suggest that some ARFs may activate transcription on a single AuxRE, but dimerization between the ARFs is necessary for transcription to occur (Lanctot et al., 2020). Enrichment for single AuxREs upstream of auxin-responsive genes has also been detectable (Freire-Rios et al., 2020), in affirmation to the yeast synthetic auxin signaling system. The biochemical mechanism underlying the differences in DNA-binding specificity of ARFs to single AuxRE binding sites is yet to be proven. Genome-wide DNA binding by ARFs has revealed both overlapping and distinct motif preferences for class A and B ARFs (Galli et al., 2018; Stigliani et al., 2019). DNA affinity purification and sequencing (DAP-seq) experiments performed on maize and Arabidopsis revealed that both class A and class B ARFs can bind IR7/8 motifs, while class A ARFs are additionally capable of binding to several DR and ER motifs (O’Malley et al., 2016; Galli et al., 2018; Stigliani et al., 2019). Although C-ARFs have been proven not to be involved in auxin-dependent transcriptional responses, at least in Marchantia (Mutte et al., 2018), one algal ARF related to the class C ARFs bind to the TGTCNN motifs (Carrillo-Carrasco et al., 2023).

Another element that determines the specificity of the DBD binding is the spacing between both sites of the AuxRE. The binding affinity of two ARFs differ significantly based on spacing between the AuxRE repeats, which dictates the formulation of a caliper model that determine specificity of ARFs binding sites (Boer et al., 2014). The dimerization ability of ARFs through their DBD or C-terminal PB1 domain permits strong binding to double-stranded DNA (dsDNA) carrying a pair of AuxREs with a spacer of a specific length (Boer et al., 2014; Pierre-Jerome et al., 2016). It has been reported that spacing of 7 or 8 bp in ARF1 and 5 to 9 bp in ARF5/MP is required between AuxRE repeats to enhance the interaction between these ARFs and their targeted AuxRE (Boer et al., 2014). Fluorophore or enzyme reporter genes under the control of synthetic promoters including DR5 promoter, characterized by tandem direct repeat of TGTCTC spaced at 5-bp intervals, has often been used for visualizing the distribution pattern of auxin signal in many plant species (Goldental-Cohen et al., 2017), suggesting that this repeat constellation is biologically meaningful.

### 2.2 Regulation of ARF activity through the C-terminal PB1 domain

The C-terminal of ARFs is a classical type -I/II PB1domain of 80-100 amino acids, which was previously named domain III/IV for ARFs and Aux/IAAs (Guilfoyle and Hagen, 2007). Besides the DBD, the PB1 domain is also an ARF interacting domain. Structural analysis on the C-terminal domain of ARFs revealed the structural basis of such heterotypic interaction of ARF5/MP (Nanao et al., 2014), ARF7 (Korasick et al., 2014), *IAA17* (Han et al., 2014), and *PsIAA4* (Dinesh et al., 2015). PB1 domains are also present in fungi, animals, amoeba, and in several protein families in plants. Characteristic of the type -I/II PB1 domains, the ARF PB1 domain permits for head to tail oligomerization, such that the positive face of one PB1 domain interacts with the negative face of another PB1 domain (Korasick et al., 2014). ARFs and Aux/IAAPB1s interact due to similarity in their 3D structure, such that one negative and one positive face will permit ARF-PB1 interact with AUX/IAA-PB1 in a head-to-tail manner through electrostatic interactions and hydrogen bonds (Vernoux et al., 2011; Piya et al., 2014). The positive face is characterized by an invariant lysine residue that interacts with an array of conserved aspartic and glutamic acids (Korasick et al., 2014), such that alteration in the lysine residue of the positive face hinders interactions with the negative face and preventing oligomerization (Powers et al., 2019).

The PB1 domain of ARFs contributes to their functioning in numerous ways. The PB1 domain mediates the interaction between ARFs and the AUX/IAA proteins, which is required for appropriate canonical auxin signal transduction, which will be discussed briefly. Mutation on the positive face of ARF19 that ablates oligomerization resulted in increased transcription of both auxin-responsive genes and novel targets in the absence of auxin (Powers et al., 2019), suggesting that the ARF19 PB1 mutant is acting as a constitutive auxin signaling factor probably due to its lack of interaction with its transcriptional corepressor Aux/IAAs. Further *in vivo* oligomerization assay revealed that ARF19 PB1 mutant did not display nuclear dimerization (Powers et al., 2019), which could be inferred that the ARF PB1 domain rather than the DBD primarily promotes ARF homodimerization. Besides the Aux/IAA-ARF interaction, the PB1 domain of ARFs is involved in transcriptional regulations. For example, ARF19 with a mutant PB1 domain that inhibits dimerization did not activate transcription of single AuxRE, but activated paired AuxRE without any hindrance. In the case of the DBD, DBD dimerization is required for both single and paired AuxRE. This data outlines the possibility that the PB1 domain confers on ARFs the ability to activate transcription of AuxREs and could stabilize ARF dimerization under less ideal AuxRE numbers.

Interestingly, the PB1 domain seems to have diverse effects on different class A ARFs, as its deletion in *Marchantia polymorpha* ARF1 generates a loss-of-function mutant (Kato et al., 2020), whereas in *A. thaliana* ARF5/MP, the mutant maintains its function and is hyperactive (Krogan et al., 2012). Although heterotypic interactions are stronger than ARF or Aux/IAA homotypic interactions, most PB1s of class A ARFs interact with Aux/IAAs. The disparity between the strength of heterotypic and homotypic interactions result from higher number of electrostatic bonds between ARF and Aux/IAA-PB1s (Parcy et al., 2016; Kim et al., 2020). However, a limited set of interactions between Aux/IAAs and Class B or C ARFs have been identified (Vernoux et al., 2011; Piya et al., 2014), which suggest that the repressor ARF proteins function independently of auxin regulation, and instead compete for DNA binding sites or heterodimerize with other ARF proteins to block transcription (Lavy et al., 2016).

### 2.3 The middle region

Between the N-terminal DBD and C-terminal of ARFs is the middle region (MR), which is highly variable among ARF TFs. Functional characterization of the middle region thus far has been quite elusive owing to its variability. Nonetheless, the middle region provides the framework for classifying the ARF family proteins. The amino acid composition of the middle region is critical in determining an ARF’s function, with glutamine-rich ARFs acting as transcriptional activators (Wu et al., 2015), whiles those enriched in serines, prolines, and threonines functioning as transcription repressors (Tiwari et al., 2003; Guilfoyle and Hagen, 2007). The activator/repressor classification correlates with the division in subgroups A/B/C, such that those ARFs tested as activators belong to class A, while class B and C ARFs encompass those tested as repressors (Tiwari et al., 2003). The activation and repressive activity of ARFs was decoupled from auxin induction by expressing the MR alone in a synthetic transcription factor assay in carrot protoplasts (Tiwari et al., 2003).

In contrast to the ARF repressor domains, the ARF activation domain remains unknown. This occurrence is probably due in part to the intrinsic disorder in the middle region of class A ARFs. Most activation domains are not characterized by semblance in their sequence, but by sequence characteristics such as hydrophobicity and negative charge (Erijman et al., 2020). It is however worth mentioning that the intrinsic disorder predominantly found in the MR of class A ARFs does not only dictate transcription potential but extends to other cellular features. For example, the MR of ARF7 and ARF19 dictates their subcellular localization (Powers et al., 2019), which is significantly influenced by the C-terminal PB1 domain. ARF19 is differentially localized to the nucleus of young roots and cytoplasm of matured roots. This tissues specific localization of ARF19 is altered by mutation in the PB1 domain, such that more ARF19 is driven to the nucleus of matured roots compared to wild-type. This cooperative relationship between ARF MR and the PB1 is believed to drive the nucleocytoplasmic partitition of ARFs through protein condensation. The PB1 domain probably increases the local concentration of ARF19 and that the intrinsic disorder of the MR contributes to phase separation and protein condensation (Powers and Strader, 2020). Just like other transcription factors, the relationship between ARF localization and transcriptional activity provides further insight into the regulatory mechanism governing the auxin signaling cascade. It is instructive to unravel the mechanisms that drive ARF condensation and the level of participation of other ARFs in this regulatory process, which will significantly broaden our understanding of auxin signaling specificity. The MR also acts as an interaction domain for the recruitment of different types of cofactors such as chromatin remodelers that aid ARFs to carry out their functions. It however remains unknown whether class B ARFs can function as transcriptional activators at certain loci or in the presence of other unknown cofactors.

Transcription activators belonging to the class A ARFs may also induce transcription indirectly by recruiting the SWITCH/SUCROSE NONFERMENTING (SWI/SNF) chromatin-remodeling complex (Clapier and Cairns, 2009). For example, the MR of *ARF5*/*MP* increases chromatin accessibility at its binding sites by recruiting the SWI/SWF complex through interactions with BRAHMA and SPLAYED, respectively (Wu et al., 2015). This result reveals a mechanism in which *ARF5*/MP, and most likely other activator ARFs, alter nucleosome positioning to make more transcription factor-binding sites accessible (Wu et al., 2015; Weijers and Wagner, 2016). In contrast, *Arabidopsis* class B ARF harbor a conserved TPL-binding motif (RLFGV), and may additionally encode a canonical ethylene-responsive element binding factor (EAR motif), which act as repressor domains *in vivo* (Choi et al., 2018). For example, both the conserved RLFGV motif and the additional EAR motif are needed for ARF2 to function as a transcriptional corepressor, but only the RLFGV motif is required for TPL interactions in yeast two-hybrid experiments (Choi et al., 2018). These evidences suggest that class B ARFs act as auxin-insensitive negative regulators of auxin-responsive genes (Kato et al., 2020). Additionally, the MR of *AtARF2* also harbors the EAR motif (Causier et al., 2012) which bears semblance to that found on Aux/IAAs and which permits interaction with the N-terminal part of TPL/TPRs (Ke et al., 2015). Class C ARFs possess a BRD-like domain with a slightly different sequence (VLFG).

## 3 The canonical and non-canonical auxin response

### 3.1 The canonical nuclear auxin pathway

**Figure 1:**
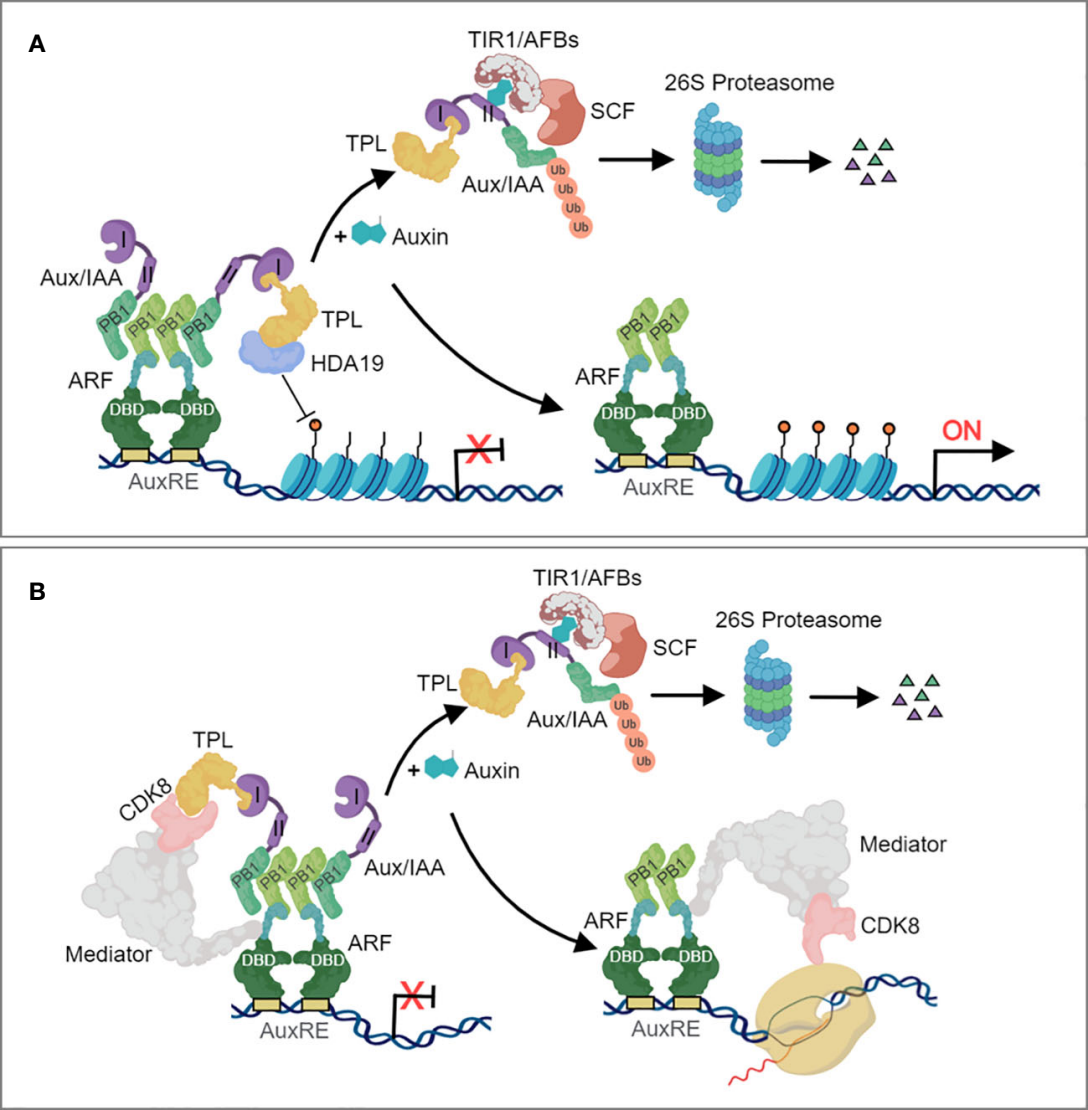
The canonical nuclear auxin signaling pathway. **(A)** Auxin signaling involving chromatin remodeling. **(B)** Auxin signaling involving the Mediator complex. In the absence of auxin, ARFs are bound by Aux/IAA repressor proteins, which recruit the TOPLESS (TPL) corepressor to constitute a repressor complex that repress transcription of auxin-responsive genes. ARFs, through their DBD domain, bind to the AuxRE of auxin-responsive genes and repress their transcriptional activity through interaction between the ARF Phox and Bem1 (PB1) domain and the Aux/IAA PB1 domain. TPL recruits histone deacetylases **(A)** and also interacts with Mediator **(B)** to prevent ARF transcriptional output. Auxin increases the affinity between the SKP1-CULLIN1-F-BOX (SCF) TIR1/AFB auxin receptor complex and Aux/IAAs, which stimulates Aux/IAA polyubiquitylation and degradation via the proteasome. Once free from TPL and Aux/IAA repression, ARFs then activates the expression of auxin-responsive genes.

Auxin regulates multiple outputs in plants primarily by controlling the activity of thousands of genes through the nuclear auxin pathway. The canonical auxin transcriptional response system was originally characterized in flowering plants. The nuclear auxin signaling pathway consists of a small number of core components which are represented by a large gene family. Changes in cellular auxin concentrations trigger transcriptional responses of numerous genes, mediated by ARF transcription factors (Weijers and Wagner, 2016). Significant advancement in understanding the auxin signaling machinery has been achieved in recent years (Weijers and Friml, 2009). The core components of the auxin signaling pathway comprises the F-box-containing Transport Inhibitor Response 1 (TIR1) and its homologous Auxin-signaling F Box Proteins (AFBs) proteins, the transcriptional co-repressors AUXIN/INDOLE-3-ACETIC ACID (Aux/IAA), and the ARF transcription factors (Wright and Nemhauser, 2015; Kong et al., 2016). Activation of gene expression as a result of IAA-mediated assembly of TIR1/AFB proteins with AUX/IAA transcriptional regulators has been accepted as the canonical auxin signalling pathway (**Figure 1****).** During auxin limitation, Aux/IAA protein binds to the C-terminal domain of ARFs and its co-repressor TOPLESS (TPL) to repress transcription. TPL recruits chromatin remodeling enzymes such as Histone Deacetylase 19 (HDA19) (**Figure 1A**) and also interacts with Mediator multiprotein complex (**Figure 1B**) to prevent ARF transcriptional output. For one case, the HDA19 acts as a physical impediment to maintain chromatin closure at the promoters of ARF-regulated auxin responsive genes (Szemenyei et al., 2008; Qiao et al., 2018) (**Figure 1A**). For another, ARFs interacts with the Mediator complex via its MR region and Aux/IAA via their PB1domains. The recruited TPL by the domain I of Aux/IAA inturn interacts with the CDK8 of the Mediator complex. Under high auxin concentration, TIR1/AFB forms SCF^TIR1/AFBs^ ubiquitin complex and triggers Aux/IAAs polyubiquitylation and degradation via the 26S proteasome, resulting in the dissociation of ARFs to TPL-HDA19 and Mediator complex. The eviction of TPL facilitates a permissive chromatin conformation and an increase in the accessibility of transcription factors on the promoters of auxin responsive genes (Wang and Estelle, 2014; Jing et al., 2015) (**Figure 1A**), and permits the ARFs-Mediator complex to recruit RNA polymerase II and leading to the initiation of gene expression (**Figure 1B**). Comparison of TIR1, AUX/IAA and ARF orthologues across land plants and charophycean algae indicate that the assembly of the canonical auxin transcriptional response pathway is a land plant innovation.

### 3.2 Non-canonical auxin–dependent signaling

**Figure 2:**
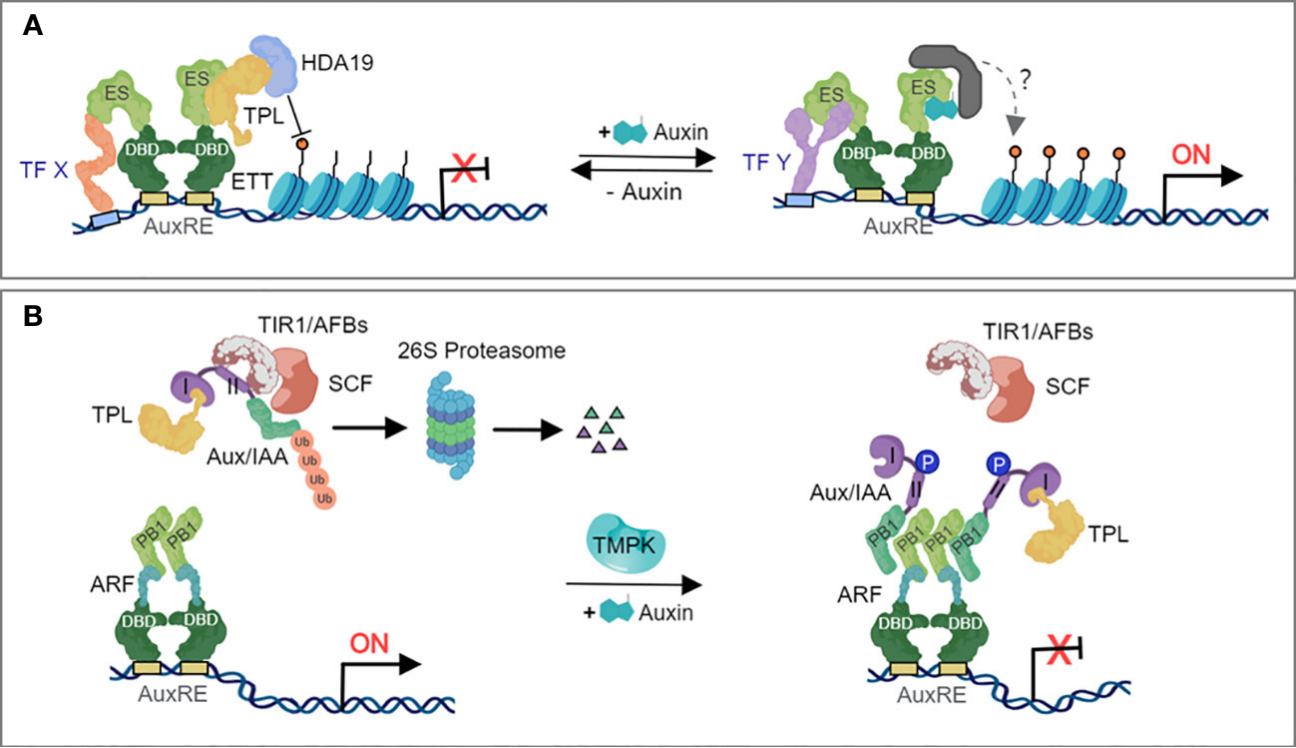
Mechanism of the non-canonical auxin-dependent signaling pathway. **(A)** The ETT-mediated non-canonical auxin signaling pathway. In the absence of auxin, the ETT-specific (ES) domain recruits the co-repressor TPL, which in turn, recruits HDA19 to deacetylate histones and repress target gene expression. Under elevated auxin levels, auxin binds to the ES domain of ETT and triggers the dissociation of the repressive complex, which releasing the repression of HDA19 and triggers histone acetylation and initiates gene expression. **(B)** The regulation of noncanonical Aux/IAAs. Certain ARFs heterodimerize with noncanonical Aux/IAAs, under no or low auxin concentrations leading to their polyubiquitylation and degradation. Auxin availability triggers phosphorylation of Aux/IAAs 4, leading to their stabilization and accumulation. These nondegradable Aux/IAAs will maintain the repression of their interactor ARFs, inhibiting the transcriptional regulation of auxin-responsive genes.

The auxin-related developmental defects of *ett* mutants suggested that ETT/ARF3 could regulate auxin signaling independently of the canonical pathway. A fundamental difference between the ETT/ARF3-mediated and the canonical models of auxin signaling is that the former does not primarily require protein degradation to activate gene expression. It was suggested that ETT/ARF3 translates local auxin concentrations to developmental outputs in the gynoecium, although the molecular mechanisms governing this occurrence had not yet been discovered (Simonini et al., 2016). ETT/ARF3 has been reported to participate in auxin dependent protein-protein interactions with several transcription factors belonging to different families, and that these interactions are relevant for auxin responsiveness of specific tissues or cell types during development (Simonini et al., 2016). In the absence of auxin, ETT/ARF3 recruits TPL to its target loci via its ES domain. TPL, in turn, recruits HDA19 to promote deacetylation of histones and repress target gene expression (**Figure 2A**). In the presence of auxin, ETT/ARF3 can directly interact with the auxin molecule via the ES domain, suggesting that binding of auxin disrupts the interaction between ETT/ARF3 and its corepressor TPL (Kuhn et al., 2020) (**Figure 2A**), which permits the regulation of auxin-responsive genes.

Another non-canonical auxin-dependent signaling mechanism involves the Trans-Membrane Protein Kinases (TMPKs) pathway. The TMPK subfamily was first linked to auxin signal transduction when the phenotypes of double, triple, and quadruple *tmpk* mutants showed cell expansion and proliferation defects, miniaturized organs, infertility, and a reduced sensitivity to exogenously applied auxin (Dai et al., 2013). Kinase cascades are rapid and could be involved in rapid, non-canonical signalling. At high auxin concentration, TMPK phosphorylates AUX/IAA at its domain II, which interferes with the poly-ubiquitination and degradation of the AUX/IAA-TPL repressor complex by the SCF^TIR1/AFBs^ ubiquitin complex, thus inhibiting the transcriptional regulation of auxin-responsive genes (**Figure 2B**).

## 4 ARF-mediated auxin pathway in growth and development

Plant growth and development are physiological processes coordinated by phytohormones. Physiological mechanisms regulating growth and development in plants are coordinated by hormonal signals, among which auxin has been implicated in virtually every aspect. Growth and development are intrinsic processes sustained by coordinated cell division, cell expansion, and cell differentiation. Auxin promotes cell division and meristem maintenance, and also plays an important role in the establishment of cellular patterning (Perrot-Rechenmann, 2010). Transcription factors are key regulators of cellular processes, both intrinsic, such as development and differentiation (Spitz and Furlong, 2012), as well as extrinsic, such as response to external signals (Lambert et al., 2018), through hormonal signaling pathways. At the molecular level, ARF TFs transduce auxin response signals by binding to the AuxRE in promoters of early auxin response genes (Wan et al., 2014). The ARFs are key components of the auxin signaling pathway known to regulate cellular processes of growth and development under normal cellular conditions (Guilfoyle and Hagen, 2007; Chandler, 2016). Several ARF genes have been reported to regulate various auxin-induced developmental processes in several plant species. ARFs are predominantly expressed during through all the periods of plant growth and development, and in different plant organs (**Table 1**), indicating its intricate role in plants.

**Table 1:** Characterization of ARFs function in various plant species.

Species	Gene name	Function	References
Arabidopsis	AtARF1/2/3	Floral organ formation	(Nishimura et al., 2005)
	AtARF1/2	*F. oxysporum* infestation response	(Lyons et al., 2015)
	AtARF2	Seed germination and primary root growth	(Wang et al., 2011)
		Lateral root growth	(Marin et al., 2010)
		Leaf flattening	(Guan et al., 2017)
		Potassium stress response regulation	(Zhao et al., 2016)
	AtARF2/ARF7	Chlorophyll accumulation	(Luo et al., 2023)
	AtARF3	Rosette leaf formation	(Schuetz et al., 2019)
	AtARF3/4	Lateral root growth	(Marin et al., 2010)
		Leaf flattening	(Guan et al., 2017)
	AtARF5	Embryonic/primary root formation	(Dastidar et al., 2019; Zhang et al., 2023)
	AtARF6/8	Adventitious root formation	(Gutierrez et al., 2009; Kou et al., 2022)
		Leaf shape/leaf reproductive organs	(Tabata et al., 2010; Xiong et al., 2021)
	AtARF8	Turnip mosaic virus response	(Jay et al., 2011)
	AtARF7/19	Lateral root formation	(Okushima et al., 2005, Okushima et al., 2007)
		Adventitious root formation	(Lee et al., 2019)
	AtARF10/16	Root cap formation	(Wang et al., 2005)
Rice	OsARF1	Crown root growth	(Waller et al., 2002)
		Leaf inclination regulation	(Xing et al., 2022)
		Somatic and reproductive tissues	(Attia et al., 2009)
	OsARF1/5/6/17/19/24/25	Nitrogen use efficiency/grain yield	(Zhang S. et al., 2021)
	OsARF3	Lemina development	(Si et al., 2022)
	OsARF4	Leaf inclination regulation	(Qiao et al., 2022)
	OsARF6/12/17/25	Flower opening and stigma size	(Zhao et al., 2022)
	OsARF11	Leaf angle regulation	(Sakamoto et al., 2013)
	OsARF12	Primary root growth	(Qi et al., 2012)
		Pi homeostasis	(Wang et al., 2014)
		Root elongation and Fe accumulation	(Qi et al., 2012)
	OsARF12/16	RDV immune response	(Qin et al., 2020)
	OsARF16	Adventitious crown root primordial formation	(Wang et al., 2007)
		Pi starvation response	(Shen et al., 2013)
		Fe-deficiency response	(Shen et al., 2013)
	OsARF17	Rice black-streaked dwarf virus response	(Zhang et al., 2020)
	OsARF19	Constitutive aerenchyma/Lateral root formation	(Yamauchi et al., 2019)
		Leaf angle regulation	(Zhang et al., 2015)
	OsARF21	Drought stress response	(Uga et al., 2013)
	OsARF23/24	Root elongation	(Li et al., 2014)
	OsARF25	Primary/crown root growth	(Mao et al., 2020)
Maize	ZmARF2/7/*25*	Maize inflorescence regulation	(Ma et al., 2023)
		Potassium uptake and homeostasis	(Sheng et al., 2020)
	ZmARF3	Leaf structure regulation	(Dotto et al., 2014)
	ZmARF4	Growth and development	(Li et al., 2022)
		Low Pi stress response	(Li et al., 2022)
	ZmARF5	Root growth and development	(Yang et al., 2022)
	ZmARf23	Embryonic callus and primary root development	(Liang et al., 2023)
	ZmARF25/35	Seminal and lateral root regulation	(von Behrens et al., 2011)
	ZmARF34	Crown root formation	(Majer et al., 2012; Xu et al., 2015)
Wheat	TaARF15	Senescence regulation	(Li et al., 2023)
Tomato	SiARF2	Salt and drought stress response	(El Mamoun et al., 2023)
	SiARF4	Drought stress response	(Chen M. et al., 2021)
	SiARF5	Fruit set and development	(Liu S. et al., 2018)
	SiARF6	Photosynthesis/sugar accumulation/fruit development	(Yuan et al., 2019)
	SiARF8/10	Salt stress response	(Bouzroud et al., 2020)
	SiARF10	Chlorophyll and sugar accumulation	(Yuan et al., 2018)
Potato	StARF10	*P. infestans* infestation response	(Natarajan et al., 2018)
	StARF16	Necrotrophic pathogen infection response	(Kalsi et al., 2022)
Poplar	PdPapARF1	*Trichoderma asperellum* infestation response	(Wang et al., 2020)
Medicago	MdARF2/3/4	Lateral Root and Nitrogen Fixing Nodule Development	(Kirolinko et al., 2021)
Betula	BpARF1	Drought stress response	(Li H. et al., 2020)
Soybean	GmARF8	Nodulation and lateral root formation	(Wang Y. et al., 2015)

### 4.1 Root morphogenesis and architecture

Plant root system plays crucial role in regulating and optimizing plant growth and development. They are important plant organs that absorb water and nutrients from soils and translocate them to the shoot (Stone et al., 2001; Sainju et al., 2005), as well as providing a means to monitor the soil for a range of environmental conditions (Overvoorde et al., 2010). Moreover, roots provide mechanical support to plants and distribute hormones that regulate numerous physiological and biochemical processes associated with growth and development of plants. Seed plants have evolved a complex root system consisting of at least three root types, i.e., the primary root, lateral roots, and adventitious roots. Since the discovery of auxins, they have been characterized to be closely related to root development. Root phenotypes associated with auxin signaling are dosage dependent, and include the length of epidermal-derived root hairs, primary root length, number and length of lateral roots and response to gravity (Ishida et al., 2008; Peret et al., 2009). ARFs have been reported to regulate various aspects of root morphogenesis and architecture in several plant species (**Table 1**).

#### 4.1.1 Arabidopsis thaliana

Primary roots develop from an embryonically formed meristem (De Smet et al., 2010) and is the first organ to emerge from a germinating seed in the form of a radicle. Among the five genes encoding *Arabidopsis* clade A ARFs, ARF5/MP is essentially involved in primary root organogenesis (Aida et al., 2002). During embryogenesis, the hypophysis acts as the primary root founder cell in Arabidopsis (Petricka et al., 2012) and requires the auxin-dependent release of MP transcription factor from its inhibition by the Aux/IAA protein BODENLOS (BDL)/IAA12 (Herud et al., 2016). MP binds directly to the AuxRE in promoter of miR390 to regulates its expression in the *A. thaliana* primary root meristem (Dastidar et al., 2019), and also controls embryonic root initiation by regulating genes that mediate signaling from embryo to hypophysis. ARF5/MP, *TARGET OF MP 5* (*TMO5*) and *TMO7* encode basic helix–loop–helix (bHLH) TFs, that are expressed in the hypophysis-adjacent embryo cells, and are required and partially sufficient for MP-dependent root initiation (Schlereth et al., 2010). Both Wuschel-related Homeobox 9 (*WOX9*) and *ARF5*/MP are required for hypophysis specification and primary root formation, with mutations in either *WOX9* or *ARF5*/MP resulting in defective stem cell niche establishment of the primary root (Breuninger et al., 2008). The WOX9-ARF5/MP complex initiates primary root formation by activating RGF1 INSENSITIVEs (RGIs) in the primary root founder cell (Zhang et al., 2023). Root cap formation in *Arabidopsis* is regulated by miRNA160, which targets ARF10 and ARF16. The Pro(35S):MIR160 and *arf10-2 arf16-2* double mutants displayed the same root tip defect, with uncontrolled cell division and blocked cell differentiation in the root distal region and showed a tumor-like root apex and loss of gravity-sensing (Wang et al., 2005). Moreover, ARF2 acted as an ABA positive responsive regulator that functions in both seed germination and primary root growth by directly regulating the expression of a homeodomain gene HB33, with ABA treatment reducing cell division and altering auxin distribution more in *arf2* mutant than in WT (Wang et al., 2011).

**Figure 3:**
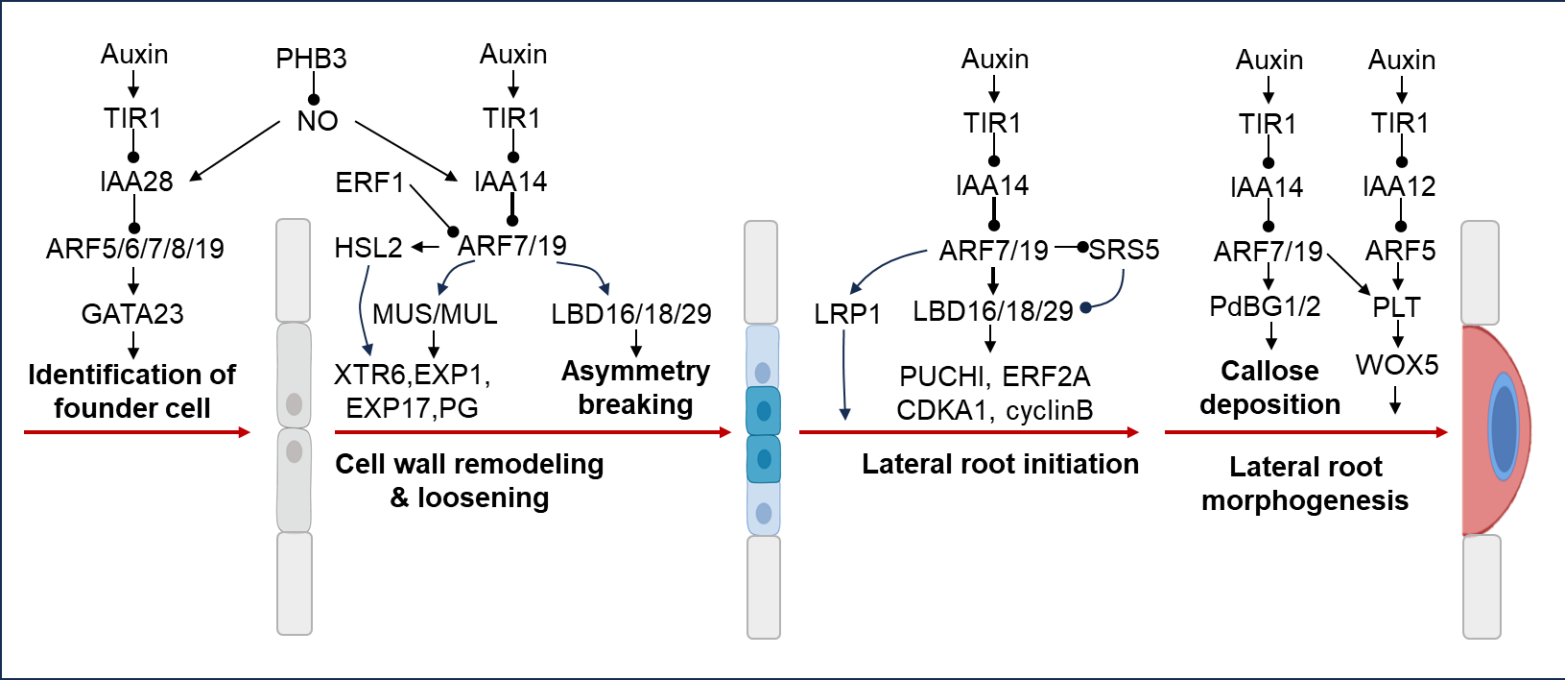
Lateral root regulation by ARFs. The IAA28-ARF5/6/7/8/19 module regulates positioning of new LRP and specification of LRFC identity by controlling the expression of GATA23 TF. PHB3 accumulates NO in pericycle cells and LRPs, which in turn triggers the degradation of AUX/IAA28/14 and the activation of ARFs and induction of *GATA23* to promote LR initiation and LRP development. ARF7 and ARF19 directly regulate the auxin-mediated transcription of *LBD16/ASL18* and/or *LBD29/ASL16* in roots and contribute to asymmetric breakage of root cell wall. Moreover, ARF7/19 regulates the expression of HSL2, MUS and MUL genes to modulate LRP initiation via regulating cell wall biosynthesis and remodeling genes such as XTR6, EXP1/17, and PGAZAT. ARF7/19 module regulates the expression of LRP1, LBD16/18/29, which inturn regulate the expression of downstream genes PUCHI, ERF2A, and CDKA1, which have been implicated in lateral root initiation. The IAA14-ARF7/19 module regulates callose deposition in LRPs during lateral root morphogenesis. ARF7/19 and ARF5/MP regulate PLETHORA 5 (PLT5), which interacts with Wuschel-related Homeobox 5 (WOX5) to regulate lateral root morphogenesis.

Lateral roots (LR) are post-embryonic roots that arise from existing roots (Atkinson et al., 2014). LRs increase the volume of soil reached by roots, provide anchorage, and participate in water and nutrient uptake and transport (Dubrovsky and Laskowski, 2017). Auxin is a crucial hormone for lateral root formation, while ARFs act as key components of auxin biosynthesis, transport, signaling, and play important roles in lateral root initiation and lateral root primordium development (Jing and Strader, 2019). The *de novo* formation of lateral root organs requires tightly coordinated asymmetric cell division of a limited number of pericycle cells located at the xylem pole. This typically involves the formation of founder cells, followed by a number of cellular changes until the cells divide and give rise to two unequally sized daughter cells. During LR initiation, a pair of xylem pole pericycle cells are primed by auxin signaling and specified as founder cells that undergo asymmetric cell division to develop as a stage I LR primordium. This process is activated by an AUX/IAA–ARF-dependent auxin signaling cascade (Luo L. et al., 2022). The module regulating founder cell formation involves the perception of auxin signaling by the auxin receptor TIR1, which acts in the basal meristem (**Figure 3**). Several Aux/IAA-ARF modules have been implicated in driving lateral root formation (Stoeckle et al., 2018). The IAA28-ARF5/6/7/19 module is specific for priming cell specification (De Smet et al., 2007; De Rybel et al., 2010), and positioning new lateral root primodia (LRP) and for specifying lateral root founder cell (LRFC) identity (Du and Scheres, 2018). Auxin-regulated GATA23 TF, considered as the first molecular marker for LRFCs, is regulated in XPP cells that leave the basal meristem by the IAA28-ARF5/6/7/19 auxin signaling cascade in the basal meristem (De Rybel et al., 2010), to regulate the process of lateral root founder cell identity (**Figure 3**). Prohibitin 3-Nitric oxide (PHB3–NO) signaling module regulates LR initiation through modulation of the canonical AUX/IAA-mediated auxin signaling cascade. PHB3 accumulates NO in pericycle cells and LRPs, and NO in turn triggers the degradation of AUX/IAA28 and IAA14 and the activation of ARFs, thereby inducing the expression of transcription factor genes *GATA23* and *Lateral organ boundaries domain 16* (*LBD16*) to promote LR initiation and LRP development (Luo L. et al., 2022). The SLR/IAA14–ARF7–ARF19 module regulates LR initiation by activating several auxin-responsive genes (Okushima et al., 2007). ARF7 and ARF19 directly regulate the auxin-mediated transcription of *LBD16/ASL18* and/or *LBD29/ASL16* in roots (Okushima et al., 2007), and contributes to asymmetric breakage of root cell wall (**Figure 3**). Auxin-dependent cell wall remodeling also has an important patterning function during LRP formation. ARF7/19 regulates the expression of Mustache (MUS) and Mustache-like (MUL) genes during LRP initiation. MUS and MUL encoding inactive LRR-RLKs, are expressed in early-stage LRPs via regulating cell wall biosynthesis and remodeling genes such as Xyloglucan Endotransglycosylase6 (XTR6), Expansin1 (EXP1), EXP17, and Polygalacturonase Abscission Zone A. Thaliana (PGAZAT) (Xun et al., 2020) (**Figure 3**). ARF7/19 also regulates *HAESA-LIKE 2* (HSL2) which is known to affect the expression of cell wall modifying and defense related genes (Niederhuth et al., 2013) (**Figure 3**). ARF7/19 module regulates the expression of LBD16/18/29, which inturn regulate the expression of downstream genes PUCHI (Goh et al., 2019), ERF2A (Berckmans et al., 2011), and CDKA1 (Feng et al., 2012), which have been implicated in lateral root initiation (**Figure 3**). ARF7/19 also regulates *Lateral Root Primordium1* (*LRP1*) (**Figure 3**), whose expression has been shown to be induced during lateral root initiation in *Arabidopsis* (Singh et al., 2020). Two callose-degrading enzymes plasmodesmal-localized β-1,3 glucanase1 (PdBG1) and PdBG2, are both transcriptionally regulated by auxin in an IAA14-ARF7/19-dependent manner, which control callose deposition in LRPs during lateral root morphogenesis (**Figure 3**). ARF7/19 and ARF5/MP regulate Plethora 5 (PLT5), which interacts with Wuschel-related Homeobox 5 (WOX5) to regulate lateral root morphogenesis.

Adventitious roots are those secondary roots that arrive from non-root tissues (Atkinson et al., 2014) whose initiation is controlled by precise balance of activator and repressor ARF transcripts, which is maintained by a complex regulatory network (Gutierrez et al., 2009). ARF6/8 are among the five genes encoding *Arabidopsis* clade A ARFs, and are required for adventitious root formation from hypocotyls (Gutierrez et al., 2009). *ARF6* and *ARF8* regulate adventitious root formation with the involvement of miRNA160 and miRNA167, such that, *ARF6* positively controls the development of adventitious roots (Kou et al., 2022). The WOX11-ARF6/8 complex activates RGIs and LBD16 to initiate the adventitious root primordium (Zhang et al., 2023). The auxin signaling module, *ARF7/ARF19-LBD16/LBD18* via AUXIN1(AUX1)/LIKE-AUXIN3 (LAX3) auxin influx carriers, is involved in adventitious root formation in *Arabidopsis*: single mutants *aux1*, *lax3*, *arf7*, *arf19*, *lbd16* and *lbd18* recorded reduced numbers of adventitious roots than in the WT (Lee et al., 2019). At the same time, double and triple mutants exhibited further decrease in adventitious root numbers compared with the corresponding single or double mutants, respectively, and the *aux1 lax3 lbd16 lbd18* quadruple mutant lacked adventitious roots.

#### 4.1.2 Rice

OsARF are large multigene family that plays essential roles in different tissues of the rice plant. OsARFs play crucial roles in modulating root developmental processes and optimal architecture of root system (RSA) essential for normal growth and development (**Table 1**). For example, *OsARF1* regulates auxin-dependent differential growth in the crown roots of rice coleoptiles, and that, *OsARF1* transcript abundance was stimulated by gravitropism in the lower fast-growing flank (Waller et al., 2002). Knockout of *OSARF12* resulted in decreased primary root length, with *osarf12* and *osarf12/25* mutants displaying shorter root elongation zone compared to WT: This was occasioned by decreased expression of auxin synthesis genes OsYUCCAs and auxin efflux carriers OsPINs and OsPGPs (Qi et al., 2012). *OsNAC2* functions as an upstream integrator of auxin and cytokinin signals by binding directly to the promoters of *OsARF25* and a cytokinin oxidase gene (*OsCKX4*) to regulate primary root length and the number of crown roots in rice (Mao et al., 2020). OsARF23/24 heterodimers binds to the promoter of an actin-binding protein (RMD) and promote its expression in the auxin signaling pathway to trigger changes in F-actin organization that controls root elongation in rice (Li et al., 2014). AUX/IAA-ARF-dependent auxin signaling controls aerenchyma and lateral root development: LR number and constitutive aerenchyma formation were reduced by the dominant-negative effect of a mutated AUX/IAA protein in the *iaa13* mutant. It was further revealed that ARF19 interacted with IAA13, and that LBD1-8 acted as a downstream target of ARF19; IAA13, ARF19, and LBD1-8 were highly expressed in the cortex and LR primordia, suggesting that these genes function in the initiation of constitutive aerenchyma and LR formation (Yamauchi et al., 2019). Rice stems develop adventitious root primordia at each node but mature slowly and eventually emerge only when the plant gets flooded (Lin and Sauter, 2018) to provide water, nutrients, and anchorage. In rice, *OsARF16* regulates the initiation of adventitious crown root primordia by activating the expression of *Crow Rootless1/Adventitious Rootless1* (*CRL1/ARL1*), which encodes an LBD protein (Liu et al., 2005; Wang et al., 2007).

#### 4.1.3 Maize

Auxin synthesis, transport and signal transduction have been proven to be involved in regulating maize root growth and development (Nestler et al., 2016). The unique roles of ARF genes in maize growth and development are emerging from molecular genetic studies (**Table 1**). Auxin signal transduction is mainly controlled by ARF and Aux/IAA genes. Multiple *AUX/IAA*-ARF-mediated signaling plays an important role in regulating plant root formation (Goh et al., 2012). *ZmIAA5* interacts with *ZmARF5* to regulate maize root growth and development. Primary root length and the number of lateral roots at the seedling stage, and total number of roots and the dry root weight at the matured stage of maize overexpressing *ZmIAA5* increased compared to the WT, while those of mutant *zmiaa5* was significantly reduced (Yang et al., 2022). Auxin has also been implicated as the starting signal that induces crown root formation in maize. Auxin induces the degradation of AUX/IAA proteins so that *ZmARF34* activate the expression of downstream target *Rootless Concerning Crown and Seminal Roots* (*RTCS*), an LOB domain protein regulating shoot-borne root initiation in maize. The induced *RTCS* proteins bind to the promoter of *ZmARF34* and activate its transcription, which inturn promotes RTCs expression, representing an amplified mutual feedback loop that regulates *ZmARF34* and *RTCS* transcription during coleoptilar node development and crown root formation in maize (Majer et al., 2012; Xu et al., 2015). The *Rootless with Undetectable Meristems 1* (*RUM1*) gene encodes *ZmIAA10* which is required for the initiation of embryonic seminal and post-embryonic lateral root initiation in primary roots of maize (Wang et al., 2010). RUM1 could interact with, and form complexes with transcriptional activators *ZmARF25* and *ZmARF34* to regulate initiation of embryonic seminal and post-embryonic lateral root initiation in primary roots of maize (von Behrens et al., 2011). *ZmARF23* bound to the promoter of a known causal gene for embryonic callus induction, *ZmSAUR15*, and positively regulated its expression at the transcription level to promote embryonic callus formation and primary root development (Liang et al., 2023).

### 4.2 Leaf regulation mechanism

Photosynthesis is crucial for the existence of the vast majority of life on earth. Plants are primary producers that form the base of every ecosystem and fuel the next tropic level by utilizing photosynthesis to transform water, sunlight and carbon dioxide into oxygen and simple energy for utilization. The photosynthetic process is the principal energy source for all organisms on earth. Leaf anatomy, such as mesophyll thickness and chloroplast abundance and distribution, influences the photosynthetic capacity of plants (Oguchi et al., 2003). Moreover, the shape, size, and chlorophyll content of plant leave influence its photosynthetic capability and efficiency (Guan et al., 2017). Auxin has been proven to play central roles in leaf developmental processes such as leaf initiation, blade formation, compound leaf patterning and leaf inclination (Xiong and Jiao, 2019), with active participation of ARFs in numerous plant species (Schuetz et al., 2019), as outlined in **Table 1**.

The flattening of leaves to form broad blades with wider surface area is a pronounced adaptation by plants to maximize photosynthetic ability and efficiency. Adaxial-expressed ARF5/MP directly binds to the promoters of *WOX1* and *Pressed Flower* (PRS) and activate their expression in the leaf marginal domain to enable leaf flattening, while redundant abaxial-enriched ARF2/ARF3/ARF4 repressors suppress *WOX1* and *PRS* expression to maintain the abaxial identity (Guan et al., 2017). While *arf3*, *arf5* and *arf7* single mutants formed normal leaves in *Arabidopsis*, *mp/arf3* or *mp/arf7* displayed a breakdown in leaf formation with novel leaf structure not present in any of the single mutants, suggesting that ARF3 and ARF7 regulates rosette leaf formation and that their functions overlap and act parallel with those of ARF5/MP (Schuetz et al., 2019). ARF6 and ARF8 activate the expression of *DWARF4* (*DWF4*), a pivotal enzyme in brassinosteroids (BR) synthesis. BRs, in turn, facilitate the demethylation of cell wall pectin, resulting in isotropic in-plane cell wall loosening, which ultimately gives rise to leaves with diverse shapes and overseeing the proximal-distal growth of leaf reproductive organs (Xiong et al., 2021). ARF2 and ARF7, with the help of IAA14, suppressed the expression of chlorophyll biosynthesis gene *Protochlorophyllide Oxidoreductase A* (*PORA*) and *Genomes Uncoupled 5* (*GUN5*) in matured leaves, resulting in reduced chloroplast number and structure in mesophyll cells and eventual reduction in photosynthetic efficiency (Luo et al., 2023).

Leaf inclination/angle is a component of crop architecture and fundamental property of plant canopy structure, which is required for light interception, canopy photosynthesis, and energy balance. Leaf inclination of rice results mainly from the asymmetric cell division and elongation of adaxial and abaxial cells at the lamina joint (Zhou et al., 2017), which is regulated by the biosynthesis or signaling of auxin. In rice, *OsARF4* participates in leaf inclination regulation via auxin and brassinosteroid (BR) signaling pathways: *osarf4* mutants displayed increase in cell differentiation on the adaxial side, resulting in increased leaf inclination; however, *OsARF4*-overexpressing lines manifested a decrease in leaf inclination, resulting in erect leaves (Qiao et al., 2022). In another experiment, *OsIAA6* interacts with *OsARF1* to suppress auxin signaling and regulates leaf inclination, with rice brassinazole resistant (*OsBZR1*), the key transcription factor in BR signaling, binding directly to the promoter of *OsIAA6* to stimulate its transcription (Xing et al., 2022), suggesting that *OsIAA6*–*OsARF1* module regulates rice leaf inclination through synergistic action of auxin and BR. The mutant *ds1* showed reduced BR sensitivity and leaf angle through a mechanism involving DS1’s interaction with *OsARF11* to regulate *OsBRI1* expression (Liu X. et al., 2018). Loss-of-function mutant of *OsARF11*, *osarf11-1*, displayed phenotypes with reduced plant height and leaf angle of flag leaves compared to WT in rice (Sakamoto et al., 2013). *OsARF19* controls rice leaf angles by positively regulating *OsGH3-5* and *OsBRI1*. *OsARF19-*overexpression rice lines showed an enlarged lamina inclination compared to WT due to its increased adaxial cell division in an auxin and brassinosteroid-dependent manner, resulting from direct activation of the early auxin responsive gene *OsGH3-1* and *Brassinosteroid Insensitive 1* (*OsBRI1*) (Zhang et al., 2015). Auxin induces *OsARF6* and *OsARF17* to independently and synergistically bind directly to the *Increased Leaf Angle1* (ILA1) promoter and activate its expression to control secondary cell wall composition of the lamina joint to determine flag leaf angle (Zhang et al., 2015).

Mutation in maize *leafbladeless1* (*lbl1*), that disrupt ta-siRNA biogenesis, give rise to plants with thread-like leaves that have lost top/bottom polarity. Misregulation of tasiR-ARFs target, ETT/*ARF3*, has emerged as the basis for the *lbl1* leaf polarity defects, with plants expressing *arf3a* transcripts displaying insensitivity to tasiR-ARF-directed cleavage and recapitulating the phenotypes observed in *lbl1* (Dotto et al., 2014). Auxin plays important roles in regulating both age-dependent and dark-induced senescence through the actions of several auxin-related genes, such as *YUCCA6*, *Small Auxin Upregulated RNA36* (*SAUR36*), and *Indole-3-acetic Acid Inducible 29* (*IAA29*) (Kim et al., 2011; Hou et al., 2013; Jiang et al., 2014). Z*mbHLH112* can repress the expression of *Aux/IAA* related genes, and promote the binding of ARF to AUXRE in the promoter of their target genes to regulate the elongation of leaf angle cells (Zhang et al., 2022).

### 4.3 Mechanism of floral structure and sexual reproduction regulation

Flowers constitute the reproductive structures in plants and lead to formation of fruit and seed after fertilization. Unlike leaves and roots that appear as single organs, flowers have evolved into a stable plant reproductive composite structure, composed of multiple organs arranged in an orderly pattern (Endress, 2010). ARFs have been reported to modulate auxin-dependent regulation of floral organ organization mostly in Arabidopsis (**Table 1**). The *ett/arf3*mutant displayed phenotypes with abnormal floral meristem patterning and gynoecium development in *Arabidopsis* (Sessions et al., 1997), whiles *arf1* and *arf2* loss-of-function mutants illustrated abnormal abscission of floral organs (Ellis et al., 2005). Mutation analyses revealed that *ARF1* and *ARF2* regulated plant leaf senescence and floral organ exfoliation, and the ETT/ARF3 gene influenced defect in pistil and flower meristem formation in *Arabidopsis thaliana* (Nishimura et al., 2005; Quint and Gray, 2006). ARF3 has been functionally characterized to participate in regulatory pathway that modulate gynoecium morphogenesis, self-incompatibility, *de novo* organ-regeneration, and organ polarity (Tantikanjana and Nasrallah, 2012). *ARF6* and *ARF8* regulated JA biosynthesis and floral organ development via suppression of class I KNOX genes *KNAT2* and *KNAT6*, with a*rf6arf8* plants displaying defective phenotypes such as aberrant vascular patterning and lack of epidermal cell differentiation in petals, which were partially suppressed by mutations in *KNAT2* or *KNAT6* (Tabata et al., 2010).

Floral organ development significantly influences plant reproduction and seed quality, yet its underlying regulatory mechanisms are still largely unknown, especially in crop plants. Disruption of *OsARF19* regulates floral organ development and plant architecture in rice. *ARF6*, *ARF12*, *ARF17*, and *ARF25*, manifested overlapping functions in flower opening and stigma size: Single mutant, *arf12*, showed a reduced plant height and aborted apical spikelets, while mutation in *ARF12* together with mutation in either *ARF6*, *ARF17*, or *ARF25* led to the same defective phenotypes including the failed elongation of stamen filaments, increased stigma size, and morphological alteration of lodicule (Zhao et al., 2022). AUX/IAA-ZmARF complexes have been reported to predominantly affect maize reproductive growth (Ori, 2019). *ZmIAA29* can influence maize florescence by interacting with *ZmARF2*, *ZmARF7*, and *ZmARF25* (Ma et al., 2023). AUX/IAA proteins Barren Inflorescence 1 and Barren Inflorescence4 and ARFs forms multiple BIF1/BIF4-ARFs transcriptional repression modules involved in the regulation of the boundary basic helix-loop-helix transcription factor *Barren Stalk1* (*BA1*), during the initial stages of reproductive organogenesis in maize and influence its inflorescence architecture (Galli et al., 2015).

## 5 The mechanism of ARFs involvement in abiotic and biotic stress responses

### 5.1 Abiotic stress

Most of the gains made towards functional characterization of ARF family proteins have focused largely on their role in plant growth and development. On the contrary, the role of auxin in regulating stress responses in plants has not received much attention. However, recent molecular approaches such as expression profiling have hinted that auxin might exert some regulatory role on plant responses to environmental stress conditions (Ha et al., 2013). It is suggested that auxin might either be acting alone or together with other key phytohormones in regulating plant response to abiotic stresses such as drought, cold, temperature extremities and salinity (Zahir et al., 2010; Lee et al., 2012). These abiotic stresses affect plant viability and development, which may result in changes in plant growth and crop yield, as well as, disturbance of physiological processes such as photosynthetic or mineral uptake rates (Kou et al., 2022). Genomic studies and expression analysis revealed that, numerous ARF family proteins were differentially expressed in various species in response to key abiotic stress such as drought, salinity or cold (Jain and Khurana, 2009), suggesting that these ARFs are active participants in abiotic stress response in plant species (**Table 1**).

#### 5.1.1 Arabidopsis thaliana

**Figure 4:**
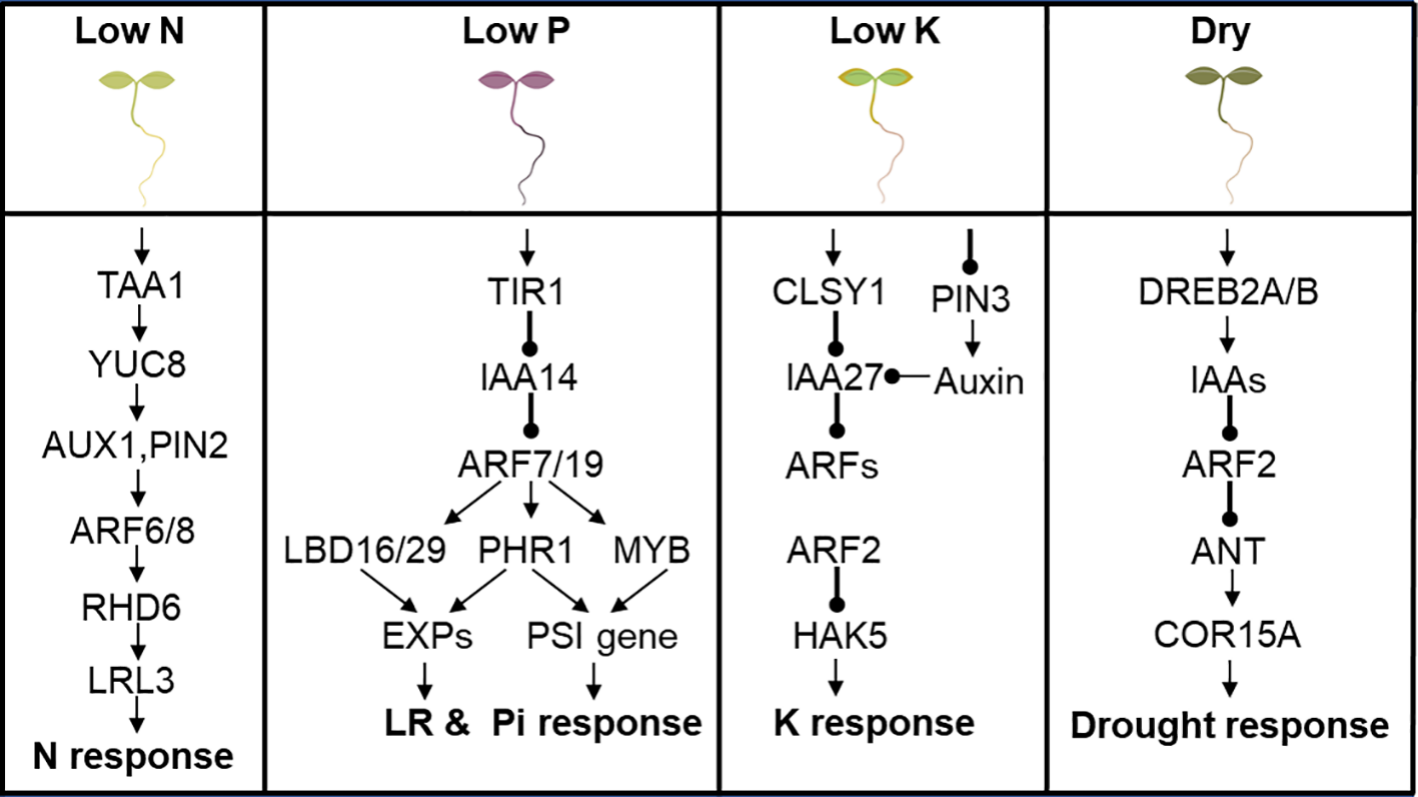
ARF is involved in abiotic stress response in Arabidopsis. **(A)** Low N upregulates TAA1 and YUCCA8 activity to regulate downstream genes ending with LRL3 to confer low N stress response. **(B)** IAA14-ARF7/19 modulates LBD16/29 and PHR1 to regulate cell wall loosening EXPs to promote lateral root development. IAA14-ARF7/19-PHR1 or IAA14-ARF7/19-MYB modulates expression of PSI genes to confer tolerance to low Pi stress. **(C)** *CLSY1* mediates the transcriptional repression of *IAA27*, an upstream regulator of ARF2, which inturn modulate the expression of the K^+^ transporter gene HAK5 and confer tolerance to low K stress. **(D)** Drought signal perception activates DREB2A/B, which directly promote transcription of IAA genes in response to drought, through a mechanism mediated by ARF2, ANT and COR15A in an ABA-dependent manner.

Nutrient deficiencies are major abiotic stresses that impact the growth, development and productivity of plants. Macronutrients are the building blocks of crucial cellular components like proteins and nucleic acids. Macronutrient deficiencies have far reaching consequence for optimum crop growth and yield optimization. Some ARFs have been implicated to participate in regulating macronutrient deficiency responses in plants. The framework of molecular components composing a cascade of auxin synthesis, transport, and signaling that triggers root hair (RH) elongation in response to low N has been proposed (Jia et al., 2023). Low N upregulates Tryptophan Aminotransferase of Arabidopsis 1 (TAA1) and YUCCA8 activities, which increase auxin accumulation in the root apex. Auxin is then translocated from the root apex to the RH differentiation zone by the auxin transport machinery comprising Auxin Transporter Protein 1 (AUX1) and Pin-formed 2 (PIN2). At the RH differentiation zone, auxin activates the transcription of ARF6/8 to stimulate epidermal and auxin-inducible transcriptional module Root Hair Defective 6 (RHD6)-Lotus Japonica Root Hairless-like 3 (LRL3) to steer RH elongation in response to low N (Jia et al., 2023) (**Figure 4**).

IAA14-ARF7/19 module has been reported to modulate LR development and confer low P stress tolerance. ARF7 and ARF19, which are transcriptional activators of early auxin response genes, acts downstream of IAA14 and regulates LR formation in Arabidopsis by directly regulating the auxin-mediated transcription of LBD16/29 in roots (Okushima et al., 2007) as shown in **Figure 4**. Auxin-responsive LBD18 acts as a specific DNA-binding transcriptional activator that directly regulates expression of Expansin (EXP) *gene*s (**Figure 4**), which encode cell wall-loosening factor that promotes lateral root emergence in *Arabidopsis thaliana* (Lee et al., 2013). PHOSPHATE STARVATION RESPONSE1 (PHR1)/MYB are recognized as key regulatory component of the response to Pi starvation by directly regulating various *P starvation-induced* (*PSI*) genes, which consequently affects P uptake and transport, and modulates RSA (Puga et al., 2017; Huang et al., 2018). ARF7 and ARF19, are the upstream regulators of the genes encoding PHR1/MYB family members (**Figure 4**).

*CLSY1*, a key component of the RNA-directed DNA-methylation machinery, mechanistically mediates the transcriptional repression of a negative regulator of root branching, *IAA27*, and promotes lateral root development under K deficiency (Shahzad et al., 2020) (**Figure 4**). IAA27 interacts with ARF2, which inturn modulates the expression of the K^+^ transporter gene HAK5 (High Affinity K^+^ transporter 5), with *arf2* mutant plants displaying a tolerant phenotype similar to the HAK5-overexpressing lines on low-K^+^ medium (Zhao et al., 2016) (**Figure 4**), and suggests that ARF2 acts as a negative regulator of low K stress response in Arabidopsis.

The molecular link that integrates plant abscisic acid (ABA) responses to drought stress in plants has been demonstrated (Meng et al., 2015). Drought signal perception leads to activation of dehydration-responsive element-binding protein (DREB2A/B) TFs which directly promote transcription of IAA genes in response to drought stress (**Figure 4**). The molecular and genetic evidence presented indicate that ARF2, ANT and Cold-regulated15A (COR15A) form an ABA-mediated signaling pathway that modulates drought stress response, with ARF2 serving as a molecular link that integrates plant ABA responses to drought stress (Meng et al., 2015) (**Figure 4**).

#### 5.1.2 Rice

Expression of seven ARF TFs, *OsARF1*, *OsARF5*, *OsARF6*, *OsARF17*, *OsARF19*, *OsARF24* and *OsARF25*, is upregulated in *dnr1* but downregulated in pAct : DNR1‐Flag overexpression line relative to WT. Upregulation of these ARF TFs mediates auxin-dependent activation of NO_3_^−^ transporter and N-metabolism genes, resulting in improved NUE and grain yield in rice (Zhang S. et al., 2021). The *osarf12* and *osarf12/25* mutants with P-intoxicated phenotypes recorded higher P concentrations, up-regulation of Pi transporter encoding genes (*OsIPS1*, *OsIPS2*, *OsSPX1*), *OsSQD2*, *OsMYB2P-1* and *OsTIR1*) and increased APase activity under Pi-sufficient/-deficient (+Pi/-Pi, 0.32/0 mM NaH_2_PO_4_) conditions compared to WT, suggesting that *OsARF12* is a negative regulator of Pi homeostasis in rice (Wang et al., 2014). Knockout of *OsARF16* led to loss of sensitivity of primary roots, lateral roots and root hairs to auxin and Pi response, with *osarf16* mutant displaying slightly reduced shoot biomass, inhibited root growth, and reduced induction of phosphate starvation-induced genes (Shen et al., 2013). Compared to WT, *osarf16* mutant displayed compromised cytokinin-induced inhibition of Pi uptake and higher Pi content under cytokinin treatment, which was occasioned by higher expression of *Phosphate Transporter1* (*PHT1*) genes, *PSI* genes and *purple PAPase* genes (Shen et al., 2014), suggesting that *OsARF16* participates in cytokinin mediated inhibition of phosphate transport and phosphate signaling in rice. Besides regulating adaptation mechanisms to macronutrient deficiencies, OsARFs have also been reported to modulate iron deficiency response adaptation in rice. *OSARF16* has been reported to regulate iron deficiency response in rice by regulating auxin redistribution: Expression of *OsARF16* is induced by Fe limitation in root and shoot, which inturn upregulates Fe-deficiency response genes; Consequently, in the auxin insensitive mutant, *osarf16*, most Fe‐deficiency symptoms were partially restored, including dwarfing, decreased photosynthesis, reduced iron content and the regulation of RSA (Shen et al., 2015). An *OsARF12* knockout mutant, *osarf12*, displayed short primary root length, altered abundance of mitochondrial iron-regulated (*OsMIR*), iron (Fe)-regulated transporter 1 (*OsIRT1*) and short postembryonic root (*OsSPR1*) in roots of rice, and resulted in limited Fe content (Qi et al., 2012).

*OsARF21* directly binds to the promoter of the early auxin responsive genes, *Deep rooting 1* (*DRO1*), and regulates its expression in the auxin signaling pathway to modulate cell elongation in the root tip, causing asymmetric root growth and downward bending of the root in response to gravity to maintain high yield performance under drought conditions (Uga et al., 2013). The rice auxin response factors, *OsARF11* and *OsARF15*, have both been reported to show differential expression under salt stress condition, suggesting that they might participate in response to salt stress response in rice (Jain and Khurana, 2009). Evaluation of changes in endogenous indole-3-acetic acid (IAA) and jasmonic acid (JA) levels and their responsive genes in rice under various abiotic stress condition revealed that *OsARF4/14/18/19* were induced by cold stress, whiles *OsARF11/13/16* were induced by heat stress (Du et al., 2013).

#### 5.1.3 Maize

Functional characterization of ZmARFs in stress response in maize remains largely limited. Nonetheless, a few ZmARF TFs have been reported to participate in stress response and adaptation. Cytonuclear localized *ZmARF2* interacts with promoter of the maize high-affinity K transporter (*ZmHAK1*) to promote K^+^ uptake and homeostasis (Sheng et al., 2020). Nucleotide diversity and favorable alleles of *ZmARF31* were found to be significantly associated with low P responses traits and root architecture in maize. Thirty, fourteen, and nine natural variations were identified in *ZmARF31* that were associated with P-deficiency-tolerance traits in maize (Wu et al., 2016). Overexpression of the maize ARF, *ZmARF4*, in Arabidopsis conferred low phosphate (Pi) stress tolerance; transgenic Arabidopsis overexpressing *ZmARF4* displayed better root development, increased Pi mobilization, up-regulation of low Pi stress inducible gene (*AtRNS1*) and down-regulation of anthocyanin biosynthesis genes (*AtDER* and *AtANS*), under low Pi stress compared to WT (Li et al., 2022).

### 5.2 Biotic stresses

Biotic stresses are those adverse conditions that normally affectplant growth due to their interaction with deleterious microorganisms such as fungi, bacteria, viruses, viroids, phytoplasmas and nematodes. These microorganisms mainly growth either on or inside plant tissues and inflict varied damages leading to symptoms like chlorosis, stunting, rotting, or local lesions formation. Compared to the role of ARF TFs in regulating responses to abiotic stresses, the role of theses TFs in biotic stress response regulation has not received much research attention. The role of auxin and its signaling pathway on plan- pathogen association has long been reported (Bari and Jones, 2009).

In *Arabidopsis*, the transcript of *ARF1* and *ARF2* were repressed by *F. oxysporum*, whiles *arf2*, *arf1* and *arf2/arf1* displayed phenotypes with increased resistance to *F. oxysporum* relative to WT, these outcomes suggest that *ARF1* and *ARF2* promote susceptibility to *F. oxysporum* infestation (Lyons et al., 2015). Misregulation of *ARF8* results in developmental abnormalities manifested by viral suppressors of RNA (VSR) transgenic plants and also for the phenotypes displayed during normal viral infection caused by the HcPro-encoding Turnip mosaic virus (TuMV) (Jay et al., 2011). Some OsARFs also play crucial roles in host antiviral immune defense. *OsARF12* and *OsARF16* interacted with *OsIAA10* to positively regulate rice antiviral defense against rice dwarf virus (RDV) through a mechanism involving binding of *OsARF12* to the AuxRE in promoter of *OsWRK1*3 to activate its transcription (Qin et al., 2020). Overexpression of *OsARF17* reduced accumulation of the black-streaked dwarf virus (BSDV) and rice black-streaked dwarf virus (RBSDV), whiles the accumulation of these virus and severity of their symptoms increased in *osarf17* knockout mutant rice lines (Zhang et al., 2020). In maize, expression of *ZmARF6* and *ZmARF18* genes increased significantly in response to *Colletotrichum graminicola* and *F. verticillioides* (Saidi and Hajibarat, 2020), suggesting that these ARFs could act as positive regulators to stresses induced by *Colletotrichum graminicola* and *F. verticillioides*.

## 6 Transcriptional and post transcriptional regulation of ARFs

**Figure 5:**
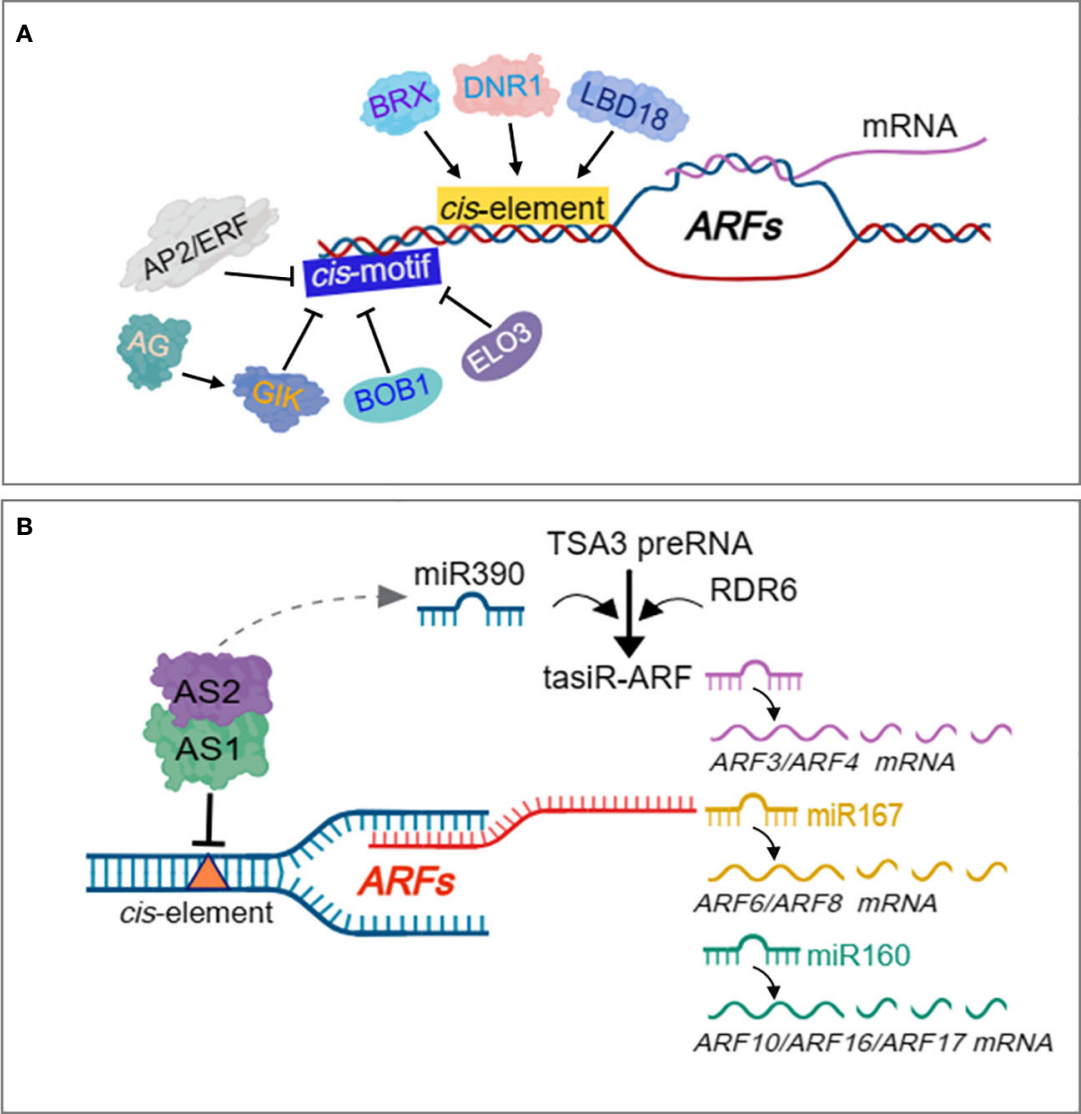
Regulation of ARFs by transcriptional and post-transcriptional events. BRX transcription co-regulator, DNR1 and LBD18, directly induce ARF expression to promote several aspects of plant growth and development. AP2/ERF, BOB1 and ELO3 directly repress expression of ARFs. AG indirectly represses ARF3 expression through GIK. AS1-AS2 complex indirectly activates miR390-and RDR6-dependent gene silencing to negatively regulate both ARF3 and ARF4 activities. The TAS3 genes encode tasiR-ARF species which target the mRNA of three ARF TFs, ARF2, ARF3/ETT and ARF4, for subsequent degradation. TAS3 harbors two miR390 target sites that are cleaved by miR138 to trigger the production of tasiR-ARF from the cleaved fragments. miRNA160 targets and cleaves *ARF10/16/17*, while miRNA167 targets and cleaves *ARF6/8* in a regulatory network that modulates adventitious root development.

ARFs have been proven to be regulated by other TFs to mediate biological process of growth and development, as well as, stress responses (Wang and Estelle, 2014). Yeast two-hybrid and *in vitro* pull down assays revealed heterodimerization between the III/IV domain of ARF5/MP and the *Arabidopsis* BREVIS RADIX (BRX) transcription co-regulator, which promotes the transactivation potential of ARF5/MP (Guilfoyle and Hagen, 2007) (**Figure 5A**), which control root meristem growth (Scacchi et al., 2010). LBD18 interacts with ARFs (**Figure 5A**) such as ARF7 and ARF19 via the Phox and BemI domains to promote the transcriptional activity of ARF7 on the AuxRE, inhibiting the negative feedback loop exerted by AUX/IAA repressor, to constitute a double positive feedback, that ensures continued lateral root growth in response to auxin in Arabidopsis (Pandey et al., 2018). A recent study showed that *Dull Nitrogen Response* TF (*DNR1*) regulates auxin homeostasis and induction of ARFs (**Figure 5A**) to promote ARF-mediated activation of *NPF*/*NRT1* and *NRT2* to regulate NO_3_^-^ uptake in roots, resulting in enhanced NUE and grain yield (Xing et al., 2023). Other regulatory models have been proposed to inhibit transcription of ARFs during growth and stress responses. For example, induction of *Agamous* (AG) represses ARF3 expression indirectly through *Giant Killer* (GIK) (**Figure 5A**) which harbors an AT-hook DNA binding motif, and is crucial for floral meristem development (Zhang et al., 2018). The *Apetala2* (APT2), encoding a putative TF characterized by a novel DNA binding motif referred to as AP2 domain, directly represses ARF3 transcription (**Figure 5A**) during floral meristem determination (Liu et al., 2014). The rice P8 proteins have been reported to interact with the C-terminus domain of *OsARF17* to prevent its dimerization with other proteins, leading to suppression of its role in conferring resistance to RBSDV and RBSD (Zhang et al., 2020). Several post-transcriptional events contribute to the cell-specific expression patterns and functions of genes. Majority of the post-transcriptional regulations of gene expression are occasioned by activities of RNA binding proteins and processing factors that are closely related with RNAs, spanning from transcription initiation to eventual death of the RNA in the cytoplasm (Dassi, 2017). MicroRNA (miRNA)-mediated regulation of auxin signaling pathway during plant development and stress responses has been reported (Luo P. et al., 2022). Numerous miRNAs have been characterized to target ARFs, leading to regulation of the downstream auxin responsive genes related to both development and stress response in plants. Two conserved miRNAs, miRNA160 and miRNA167, constitutes a complex feedback loop that regulates processes in the auxin signaling pathway by modulating the expression of ARFs (Singh and Singh, 2021)(**Figure 5B**). The miRNA160 and miRNA167 actively regulate mRNA abundance of ARFs in Arabidopsis, miRNA160 targets and cleaves *ARF10/16/17*, while miRNA167 targets and cleaves *ARF6/8* (Mallory et al., 2005; Wu et al., 2006) (**Figure 5B**). The miRNA160/miRNA167 and their associated targets *ARF6/8/17* form a regulatory network that modulates adventitious root development. Whiles miRNA167 targets ARF6 and ARF8, which functions as positive regulators of adventitious root development, miRNA160 targets ARF17, which acts as a negative regulator of adventitious root development (Gutierrez et al., 2009). However, *ARF6/8/17* control their own expression at both transcriptional and posttranscriptional level by regulating the abundance of miRNA160 and miRNA167, which completes the miRNA160/miRNA167-*AtARF6/8/17* feedback loop that regulates adventitious root development (Gutierrez et al., 2009). OsmiRi167a targets *OsARF12*, *OsARF17* and *OsARF25* to control tiller angle in rice, with repression of *OsARF12*, *OsARF17* and *OsARF25* in transgenic plants overexpressing OsmiRi167a, which displayed phenotypes with larger tiller angle similar to *osarf12/osarf17* and *osarf12/osarf25* plants (Li Y. et al., 2020). The miRNA167a positively regulates grain length and weight by dictating *OsARF6* mRNA silencing to mediate *OsAUX3* expression in a novel *miRNA167a-OsARF6-OsAUX3* regulatory model (Qiao et al., 2021). The miRNA160 has also been reported to target ARF10 and ARF16, which act as transcription repressors, and regulate the expression of their downstream responsive genes to mediate the regulation of developmental processes in plants (Huang et al., 2016; Liu et al., 2016).

The most well studied Trans-acting SIRNA (TAS)-derived short interfering RNAs (siRNAs) are tasiR-ARFs, which are widely conserved across plant species and target several ARF genes (Allen et al., 2005). The TAS3 genes encode tasiR-ARF species which target the mRNA of three ARF TFs, ARF2, ETT/ARF3 and ARF4, for subsequent degradation (Ozerova et al., 2013) (**Figure 5B**). miRNA can trigger the biogenesis of secondary siRNAs in phase (phasiRNAs) such as the TAS by targeting their transcripts for cleavage (Liu et al., 2020). The cleaved TAS transcripts is bound to and converted to double-stranded RNAs (dsRNAs) by RNA-binding protein SUPPRSSOR OF GENE SILENCING 3 (SGS3), through RNA-dependent RNA polymerase (RDR6), and undergoes further processing to generate phasiRNAs such as tasiR-ARF (Zhang et al., 2019) (**Figure 5B**). In another mechanism, TAS RNA precursor TAS3 transcript bears two targets sites of miR390, cleavage at these sites trigger the production of phasiRNAs such as tasiR-ARF from the cleaved fragments (Axtell et al., 2006) (**Figure 5B**). ARF2, ARF3 and ARF4 have been demonstrated to be targeted and regulated by TAS3 ta-siRNA (tasiRNA-ARF) (Hunter et al., 2006) (**Figure 5B**), which affects developmental timing and patterning in *Arabidopsis* (Fahlgren et al., 2006). Assymetric leaves 1 (AS1)-AS2 also indirectly activates miR390-and RDR6-dependent post-transcriptional gene silencing to negatively regulate both ARF3 and ARF4 activities (Iwasaki et al., 2013) (**Figure 5B**).

miR167 positively regulates nodulation and lateral root development in *Glycine max* by targeting and inhibiting its target genes *GmARF8a* and *GmARF8b* (Wang Y. et al., 2015). miR167 has also been reported to positively regulate plant development and root plasticity by targetingARF6 and Indole acetic acid alanine resistant3 (IAR3) (Kinoshita et al., 2012). Digital gene expression profile revealed that microRNA response element, miRNA167, targets *TcARF6* to constitute a tch-miRNA167-TcARF6 negative response module that downregulates the expression of *TcARF6* in roots of *Tamarix chinensis* in response to salt stress (Ye et al., 2020). The expression of miRNA160a/b was strongly upregulated whiles their target ARF10 was downregulated in two cowpea genotypes under drought stress treatment (Barrera-Figueroa et al., 2011). Analysis of ta-siRNA synthesis mutants and mutated *ARF3*-overexpressing plants that escape tasiRNA-ARF targeting indicated that, self-pollination was hampered by short stamens in plants under drought and high salinity stress, suggesting that tasiRNA-ARF is involved in maintaining the normal morphogenesis of flowers in plants under drought and high salinity stress conditions (Matsui et al., 2014). Salt stress treatment (100 mM NaCl) induced expression of miR390, increased cleavage of TAS3, produced higher levels of tasiARFs, and subsequently enhanced cleavage of ARF3/4 (Wen et al., 2020). A miRNA160-ARF regulatory network modulates male sterility caused by long exposure to high temperature stress: overexpression of miRNA160 increased sensitivity of cotton to high temperature stress, with a reduction in ARF10/17 mRNA, leading to activation of the auxin response at the sporogenous cell proliferation stage (Ding et al., 2017; Chen et al., 2020). *NtmiRNA167a* transcriptionally regulates *NtARF6* and *NtARF8* to mediate drastic plant Pi-starvation response via modulation of various biological processes in a miRNA167a-ARF6-ARF8 negative response regulatory module, where *NtmiRNA167a* overexpression and *NtARF6* knockdown mutant displayed reduced plant growth, biomass and increased ROS accumulation under Pi-starvation condition compared to WT (Chen et al., 2018). Interaction between miRNA160 and miRNA165/166 modulates numerous downstream responsive biological processes, in which ARFs and HD-ZIP IIIs play opposite roles in regulating leaf development and drought stress response (Yang et al., 2019). The miRNA167-ARF8 regulatory module has been revealed to regulate cell type-specific response to available nitrogen status and plastic development of lateral roots in Arabidopsis (Gifford et al., 2008).

Analysis of differentially expressed miRNA target genes revealed that, miRNA160 was induced by bacterial and fungal pathogen infection, whiles its ARF target genes were downregulated in a miRNA160-ARF module, which regulated defense response in Arabidopsis against *Botrytis cinerea* (Xue and Yi, 2018), in banana against *Fusarium oxysporum* (Cheng et al., 2019) and in cassava against *Colletotrichum gloeosporioides* (Pinweha et al., 2015). AtmiRNA167a targets the transcription of ARF6 and ARF8 to regulate the closure of leaf stomata to prevent entry of *Pseudomonas syringae*, with P_35S_:MIRNA167a overexpression and *arf6-2 arf8-3* plants displaying extreme resistant phenotypes compared to WT (Caruana et al., 2020). The miRNA390-tasiRNA-ARF regulatory module regulates lateral root development under salt stress, with significant inhibition in expression of ARF3.1, ARF3.2 and ARF4 in miRNA390-overexpressing line under salt stress but increased in the miRNA390-knockout line (He et al., 2018).

## 7 Regulation of ARFs by epigenetic modes

Epigenetic mechanisms play crucial roles during the life cycle of living organisms (Duan et al., 2018), which help cells to control gene activity without changing the DNA sequence. These mechanisms help determine whether specific genes are tuned on or off, and ensure that each cell produces only the proteins that are necessary for its function (Gayon, 2016). The three most prominent epigenetic mechanisms are histone modification, DNA methylation, and noncoding RNA (ncRNA) regulation (Fessele and Wright, 2018). ARF-dependent induction of downstream auxin-responsive genes is regulated by multiple epigenetic factors, including histone modifications and the chromatin remodeling factor PICKLE (PKL) (Weiste and Droge-Laser, 2014).

### 7.1 Histone modifications

Histone acetylation is a key histone modification mechanism that appears to be a dynamic reversible switch for inter-conversion between permissive and repressive transcriptional states of chromatin domains (Zhou and Hu, 2010). The co-repressor TOPLESS (TPL) recruits *HDA19* to the auxin signaling repressor, AUX/IAA, in an EAR motif-dependent manner, and that the function of GCN5/HAG1 histone acetylase is directly opposed to the function of *IAA12/BDL-TPL-HDA19* repressor complex in the ARF-dependent expression of auxin responsive genes (Long et al., 2006; Szemenyei et al., 2008). ARF18-HISTONEDEACETYLASE6 (HDA6) module regulates floral organ identity in *Rosa hybrid*: Silencing of *RhHAD6* increases H3K9/K14 acetylation levels at the site adjacent to the *RhARF18*-binding site in the promoter of its downstream target, *RhAG*, and reduces petal number (Chen J. et al., 2021), which indicates that *RhARF18* probably recruit *RhHDA6* to the *RhAG* promoter to repress *RhAG* transcription.

### 7.2 DNA methylation

DNA methylation is one of the prominent epigenetic modifications that occur extensively in living organisms (Wang et al., 2009). DNA methylation causes changes in chromatin state in plant cells undergoing dedifferentiation (Koukalova et al., 2005), and can also help to establish or maintain the undifferentiated cell state in plants (Berdasco et al., 2008). In plants, DNA demethylation depends on four bifunctional 5-methylcytosine glycosylases: Repressor of silencing 1 (ROS1), Demeter (DME), DME-like 2 (DML2), and DML3, which remove methylated bases and cleave the DNA backbone at abasic sites. The increased expression of *AUXIN RESPONSE FACTOR3 (ARF3)* in *met1* indeed was due to DNA demethylation, suggesting that DNA methylation regulates *de novo* shoot regeneration by modulating auxin signaling (Li et al., 2011). BOBBERY1 (BOB1), an Arabidopsis orthologue of eukaryotic NudC domain proteins, and ELONGATA3 (ELO3), the catalytic subunit of the hioghly conserved elongator complex in Arabidopsis, has been revealed through genetic analysis to repress expression of ARF3 and ARF4, along with AS1-AS2 (Takahashi et al., 2013). BIN2 has been reported to phosphorylate ARF7 and ARF19, and in contrast to reducing activity of ARF2, ARF7, and ARF19 phosphorylation enhanced their transactivation activity, which is attributed to reduced ARF7 and ARF19 interactions with the Aux/IAA repressors.

## 8 Post-translational regulation of ARFs

Post-translational regulation refers to those cellular events that regulate the abundance of active proteins. It predominantly occurs either by means of reversible events as evident through post-translational modifications (PTMs) or through irreversible events such as proteolysis. PTMs are covalent processing activities that modify the properties of active proteins via proteolytic cleavage and addition of modifying group such as acetyl, phosphoryl, glycosyl and methyl, to one or multiple amino acids (Ramazi et al., 2020).

### 8.1 Phosphorylation

Protein phosphorylation is the most prominent PTM that acts as a crucial cellular regulatory mechanism to either activate or deactivate enzymes and receptors by phosphorylation or dephosphorylation events, which are respectively catalyzed by kinases and phosphatases at serine, threonine, or tyrosine residues (Ardito et al., 2017). The mitogen-activated protein kinases (MAPK) cascades are conserved signaling mechanism comprising reversible phosphorylation through a cascade of ATP-dependent protein kinases, which regulates multiple aspects of plant growth and development. It has been proposed that auxin signal transduction is mediated by the conserved MAPK signaling cascade (Mizoguchi et al., 1994). Auxin-induced MPK14 phosphorylated and stabilized non-canonical IAA33 and enhanced its competitiveness over canonical repressor IAA5 for binding site on promoters of ARF10 and ARF16, which mitigated inhibition of ARF10 and ARF16 by IAA5 and promoted the identity of root distal stem cell (DSC) and negatively regulated auxin signaling (Lv et al., 2020). In the canonical NAP, drought-induced MPK3/MPK6 phosphorylates and stabilizes IAA15 by inhibiting TIR1-mediated ubiquitination of IAA15, which inturn represses the transcriptional activation of LBD genes by ARF7 and ARF19, leading to limited lateral root development under drought stress in Arabidopsis (Kim et al., 2022). In the non-canonical NAP, some TMK1 family members function as PM-resident receptors or part of a receptor complex, perceiving extracellular auxin and transducing these signals into various phosphorylation events (Tan et al., 2021). The cytosolic and nucleus-translocated C terminus of TMK1 specifically interacts with and phosphorylates non-canonical IAA32 and IAA34 repressors of auxin signaling, thereby regulating ARF transcription factors to dictate differential growth of the apical hook (Cao et al., 2019). Other phosphorylation events have been reported to regulate ARF protein functions. For example, ARF2 mostly represses the expression of the HAK5 potassium transporter gene, meanwhile ARF2 is phosphorylated under low potassium stress to abolish its ability to bind to the promoter of HAK5 and diminishes its repressive effects on HAK5 (Zhao et al., 2016). The BRASSINOSTEROID-INSENSITIVE 2 (BIN2) has been implicated to target and phosphorylate ARF7 and ARF19: Phosphorylation of ARF7 and ARF19 suppresses their interaction the AUX/IAA repressor, which eventually enhances the transcription activity of ARF7 and ARF19 to regulate their downstream target gene *LBD16* and *LBD29* to promote lateral root organogenesis (Cho et al., 2014).

### 8.2 Ubiquitination

Previous studies on the role of proteolytic regulation in auxin signaling have focused on degradation of their interacting partner, the Aux/IAA proteins, as described above. Although ARF proteins have been shown to be degraded through the 26S mediated ubiquitination, and the degradation process occurs independent of IAAs (Salmon et al., 2008), not much data has been generated regarding degradation of ARFs. Degradation analysis in ARFs show that 37°C treatment increased the protein levels of *HA-ARF5*/MP, *HA-ARF6*, and *HA-ARF10*. On the contrary, there was a pronounced reduction in protein levels of these *HA-ARF*s by ABA, 4°C and salt treatments, whiles MG132 inhibited the reduction of *HA-ARF6* level by ABA and 4°C treatments, suggesting that the *ARF* protein levels are regulated by multiple factors and that these treatments decrease *HA-ARF6* level through 26S proteasome-mediated protein degradation (Li K. et al., 2020). MG132 suppressed the ethylene-dependent decrease in ARF2 protein levels during apical hook development, which strongly suggests that the ethylene-mediated degradation of ARF2 protein is via 26S proteasome degradation pathway (Li et al., 2004). Functional characterization of F-box protein AUXIN RESPONSE FACTOR F-BOX1 (ARF1) SCF^ARF1^) revealed that this E3 ubiquitin ligase directly interacts with ARF7 and ARF19 to promote their degradation, and regulate their accumulation, condensation, and nucleo-cytoplasmic partitioning, which triggers downstream auxin responses (Jing et al., 2022).

### 8.3 SUMO modification

Small ubiquitin-like modifier (SUMO) is emerging as an important posttranslational modification that regulates plant development and defense pathways (Orosa-Puente et al., 2018; Niu et al., 2019). SUMO is covalently attached to the lysine residues of target proteins, which could modulate protein activity, stability, localization, and protein-protein interactions of target proteins (Vierstra, 2009), converse to protein degradation as witnessed during ubiquitination. SUMOylation is a crucial PTM that has significantly affected various plant responses to stress and environmental changes (Benlloch and Lois, 2018). MdARF8 is directly SUMOylated by apple SUMO E3 ligase MdSIZ1, which enhances protein stability of MdARF8, and facilitates LR formation in apple (Zhang C. L. et al., 2021). The uneven distribution of water in the soil has a direct influence on plant growth and root architecture, which are regulated by the SUMOylation of ARF7 (Bao et al., 2014; Orosa-Puente et al., 2018). SUMOylated ARF7 enhances its binding capacity to IAA3 and negatively regulates ARF7 activity, thereby inhibiting the expression of LBD16 (Orosa-Puente et al., 2018). Conversely, nonSUMOylated ARF7 cannot recruit IAA3 on the moisture side, which leads to an increased expression of LBD16 and promoted LR development (Orosa-Puente et al., 2018).

## 9 Conclusions and perspectives

Over the past decades, the auxin signaling pathway has emerged as a complex regulatory system that modulates plant growth, development, and stresses response. ARF transcription factors serve as effectors of auxin response that transduce and translate auxin signals into the regulation of auxin responsive genes. Both forward and reverse genetic approaches have deepened our understanding of the influence of ARFs on plant development and stress responses. The differential expression of various ARFs in response to various abiotic and biotic stresses suggests that ARFs might exhibit overlapping regulatory roles in response to these stresses. We have also reviewed the modulation of ARF expression by other molecular regulators and how these transcriptional regulations influence the role of ARFs in stress response in plants.

So far, studies on ARF TFs have primarily emphasized on gene cloning and functional characterization, with majority of them focusing transcriptional levels where ARFs bind to cis-acting elements in promoter of their target genes to regulate their expression. Comparably, research on post-translational modification of ARF TFs, including mRNA precursor splicing, editing, stability, nuclear transport, and siRNA-mediated modification—critical for stress response regulation in plants—remains very limited. We propose that future analyses of ARF TFs should emphasize the synergy between transcription regulatory factors, post-transcriptional and post-translational modifications, with a strong focus on the mechanisms of action governing the post-translational modifications of ARF TFs.

Moreover, studies on ARFs have predominantly concentrated on the function of individual ARF TFs or their interaction with other proteins. However, the mechanism governing ARFs function is highly complex due to the larger number of the ARF TF family members and the scattered nature of recent research. Consequently, the regulatory network of ARF TFs remains poorly understood. Further exploration and investigation are needed to understand the role of ARF TFs in perception and transduction of internal and external signals and the interaction among various ARF TFs on physiological and biochemical processes.

It is also important to note that, although plants often encounter multiple stresses, most ARF research has focused on the function of ARFs under single stress conditions. Future functional characterization of ARF TFs should include analyses in response to multiple stresses, followed by comparison of the differences and similarities between single and multiple stresses conditions. This approach is expected to identify key nodes in the complex regulatory network of ARFs. It is also worth highlighting that many datasets related to ARF TFs are scattered and requires integration into a specific online database, which will enable researchers to access relevant ARF TF information quickly.

Research findings have revealed the potential of ARFs in regulating multiple stress conditions, highlighting the functional complexity of ARFs and emphasizing the need to address all aspects of their functioning. Recent studies have shown the existence of crosstalk in some ARF TFs, and ARFs exert their function through various signaling pathways, which can be influenced by both crosstalk and mutual coordination mechanisms.

## Conflicts of interest

The authors declare that the research was conducted in the absence of any commercial or financial relationships that could be construed as a potential conflict of interest.

## Author contributions

LL: Funding acquisition, Writing – review & editing, Conceptualization, Data curation, Investigation, Writing – original draft. BY: Writing – original draft, Writing – review & editing, Data curation, Investigation. JL: Writing – review & editing, Formal analysis. FW: Conceptualization, Funding acquisition, Supervision, Writing – review & editing.
